# *Foxf1*-mediated co-regulation of *miR-495* and *let-7c* modulates epicardial cell migration and myocardial specification

**DOI:** 10.1007/s00018-025-05735-4

**Published:** 2025-06-25

**Authors:** Juan Manuel Castillo-Casas, Ángel Dueñas, Francisco Hernández-Torres, Rita Carmona, Ramón Muñoz-Chápuli, Ana Dopazo, Rebeca Álvarez, Enrique Vázquez de Luis, Amelia E. Aranega, Diego Franco, Estefanía Lozano-Velasco

**Affiliations:** 1 Cardiovascular Development Group, Department of Experimental Biology, Faculty of Experimental Sciences, University of Jaén, 23071 Jaén, Spain; 2 Fundación Medina, 18016 Granada, Spain; 3 Department of Biochemistry, Molecular Biology III and Immunology, Faculty of Medicine, 18016 Granada, Spain; 4 Department of Animal Biology, University of Málaga, 29071 Málaga, Spain; 5 Department of Human Anatomy, Legal Medicine and History of Science, Faculty of Medicine, University of Málaga, 29071 Málaga, Spain; 6 Genomic Unit, Centro Nacional de Investigaciones Cardiovasculares, 28029 Madrid, Spain; 7 CIBER de Enfermedades Cardiovasculares (CIBERCV), 28029 Madrid, Spain

**Keywords:** Transcription factors, MicroRNAs, Epicardial cells, Cell migration, Cell lineage specification

## Abstract

**Background:**

The heart is the first functional organ to develop in the vertebrate embryos. In mice, the primitive tubular heart begins beating at embryonic day (E) 8.0-E.8.5 and undergoes rightward looping to form the atrial and ventricular chambers. The proepicardium, a transient cell cluster at the sinus venous-lateral plate mesenchyme junction migrates onto the heart and gives rise to the embryonic epicardium, a squamous epithelium that plays a key role in cardiac development. Despite advances in understanding epicardial lineage contributions, the molecular mechanisms governing these processes remain poorly understood.

**Methods:**

To characterize the transcriptional and post-transcriptional regulation of epicardial development, we performed RNA sequencing at two critical timepoints, proepicardium formation and embryonic epicardium establishment. We analysed differentially expressed coding and non-coding RNAs, focusing on microRNAs and their potential regulatory interactions.

**Results:**

We identified a complex network involving differentially expressed mRNAs, microRNAs and lncRNAs between proepicardium and embryonic epicardium. Notably, with *miR-495 and let-7c* emerged as key regulators of epicardial cell migration, an essential process for proper epicardium formation and epicardial-derived cell migration. Our findings also reveal that these microRNAs not only regulate target gene expression but also modulate other microRNAs, suggesting a novel regulatory mechanism in epicardial development. Additionally, *Foxf1* inhibition modulates *let-7c,* promoting the expression of key cardiogenic lineage markers in epicardial cells.

**Conclusion:**

Our study highlights the role of *Foxf1* in regulating *miR-495* and *let-7c*, which in turn modulate epicardial cell migration and myocardial specification. These finding provide new insights into the intricate interplay between transcription factors and microRNAs in governing cardiogenesis.

**Supplementary Information:**

The online version contains supplementary material available at 10.1007/s00018-025-05735-4.

## Background

The heart is the first organ that becomes functional in the vertebrate embryo. In mice, the precardiac mesoderm forms a primitive tubular heart, beating at embryonic day (E) 8.0-E.8.5. This tubular structure undergoes a series of morphological changes, including rightward looping and thereafter configuring the prospective atrial and ventricular chambers (E9.5) [1]. At E10.5, five distinct regions can be delineated in the embryonic heart, the inflow tract, the embryonic atrial chamber, the atrioventricular canal, the ventricular chambers and the outflow tract [2]. From this stage onwards, each embryonic cardiac region will be separated into distinct left and right components, providing thus a double circuitry with distinct inlet and outlet connections [1]. Besides the intrinsic cardiac progenitor cells, external cell populations also contribute to heart development i.e. the cardiac neural crest and the proepicardium and its derivatives [3, 4]. The proepicardium (PE) is a transitory cell cluster that develops at the junction between the sinus venosus (SV) and the posterior undifferentiated lateral plate mesenchyme (LPM) at E8.5-E9.0 in the mouse embryo [5, 6]. In chicken embryos, PE grows in size and villous projections extend towards the dorsal aspect of the cardiac inner curvature, ultimately contacting the atrioventricular (AV) junction and forming a tissue bridge [7]. In mice, around E10.0 proepicardial villous projections attach to the heart [6] and proepicardial cells migrate and spread over the naked myocardium forming a single squamous epithelium which is termed the embryonic epicardium (EE), playing an essential role in cardiac development [5, 8].

The epicardium, the outermost layer of the heart, was long considered as an external cover devoid of any functional meaning, but recent studies discovered its essential contribution to the cardiac development and regeneration [9–12]. The EE serves as a crucial source of epicardial-derived cells (EPDCs) that, after undergoing epithelial-to-mesenchymal transformation (EMT), migrate into the myocardial wall and differentiate into multiple cardiac cell types [13, 14]. EPDCs contribute to endothelial and smooth muscle cells in the coronary vasculature, cardiac fibroblasts [15–17], and to a lesser extent, atrioventricular cushion cells [18–20]. More recently, a contribution to cardiac resident stem cells (mesenchymal-like) has also been reported [21] as well as to the cardiomyocyte lineage [22–24], yet this latter point remains highly controversial [25, 26]. Recent studies have enhanced our understanding of the molecular mechanisms driving PE and EE tissue formation [26]. Signaling molecules such as Bmp and Fgf play pivotal roles in PE specification and cardiomyogenic differentiation [27]. Transcription factors such as *Wt1* are crucial for EMT and EPDCs maturation [11, 28–32]. *Tbx18* has a role in epicardial EMT and subsequently in differentiation of EPDCs into smooth muscle cells and fibroblasts [33, 34] while *Tcf21* regulates proepicardial cell specification and maturation [35]. Finally, while *Gata4* is essential for PE formation, the precise contributions of other cardiac-enriched transcription factors such as *Nkx2.5*, *Isl1*, and *Pitx2* remain unclear [36–38]. Despite these advances, it is poorly understood how transcription factors and non-coding RNAs contribute to epicardial development.

While transcriptional regulation plays a critical role in cardiac morphogenesis and cardiovascular cell differentiation, a growing body of evidence suggests that microRNAs, the most studied subtype of small non-coding RNAs, play crucial roles in gene regulation during embryonic development and tissue homeostasis [39–42]. microRNAs display temporal and spatial differential expression in both embryonic and adult tissues, where they fine-tune gene expression at the post-transcriptional level [43]. In the context of cardiogenesis, several microRNAs have been implicated in cardiac differentiation, proliferation and morphogenesis [44–48]. Recent studies in our laboratory evidenced a microRNA differential expression during PE and EE formation in chicken, identifying *miR-146*, *miR-195* and *miR-223* as potential regulators that selectively enhance cardiomyogenesis in PE and EE by modulating *Smad3* and *Smurf1*, in ex vivo conditions [48]. Considering the species-specific differences in epicardium formation and the discovery of DE microRNAs in chicken, the functional role of microRNAs in PE and EE development in mice, as well as their potential application to enhance cardiogenesis remains elusive.

Although significant progress has been made in understanding the cell lineage contribution of the EPDCs over the last decade, the molecular determinants that contribute to such cell fate decisions remains largely unknown. In this study, we carried out a comprehensive RNAseq analysis of coding and non-coding gene expression at two critical timepoints of PE and EE development in mouse embryos. Our data identified an intricate network of differentially expressed (DE) mRNAs, microRNAs and lncRNAs that regulate distinct biological pathways in PE *vs*. EE. We identified that *Foxf1* transcription factor exerts a regulatory control over *miR-495, miR-351,* and *let-7c*, thereby modulating epicardial cell migration and myocardial specification. These observations underscores the complex interplay between transcription factors and microRNAs in epicardial development, providing new insights into the molecular mechanisms that govern cardiogenesis during embryonic development.

## Methods

A comprehensive description of each procedure is detailed in the following sections. Supplementary Fig. 1 provides an overview of the experimental workflow, illustrating the key methodological steps.

### Mouse lines and tissue collection

Previously described Wt1^GFP/+^ mice were used in this study. The WT1^GFP^ knockin line in which the exon 1 of a Wt1 allele has been replaced by the GFP sequence was used as a reporter for active WT1 transcription [49]. Pregnant Wt1^GFP/+^ female mice were harvested to E9.5 and to E10.5, respectively. E9.5 PE were manually dissected, pooled and stored in buffer lysis for RNA isolation at −80°C until used. For flow cytometry analysis and sorting, dissected hearts from E10.5 embryos were placed in cytometry buffer (phosphate buffer saline [PBS] plus 2% fetal bovine serum [FBS] and 10 mM 4-(2-hydroxyethyl)- 1-piperazineethanesulfonic acid [HEPES]) and homogenized by repeated pipetting. Cell suspension was washed by pelleting at 400G during 5 min. Then, cells were incubated on ice in darkness with the fluorochrome conjugated antibodies: anti-CD31-APC (PECAM-1) (Mouse monoclonal anti-CD31 APC #Thermo Fisher, 17–0311-82) for general staining of the endothelium. 4',6-Diamidine-2'-phenylindole dihydrochloride (DAPI) staining was included to exclude dead cells. Negative controls (GFP negative littermates) and isotypic antibody allowed setting of the gates (FITC Rat IgG2a, k Isotype, #Biolegend). Epicardial cells were then sorted by GFP high fluorescence and lack of CD31 staining (Supplementary Fig. 2) [50]. Cells were sorted in a BD FACS Aria Fusion Cell Sorter. Data were analyzed with FlowJo TM10. After sorting, epicardial cells were pooled and stored in buffer lysis for RNA isolation at −80°C until used. At least 3–5 litters were used at each developmental stage until sufficient tissue was collected, which would guarantee optimal and sufficient RNA isolation for further sequencing.

### RNA-seq libraries preparation, sequencing and differential expression gene analysis

Total RNA from PE and EE samples was extracted by using RNAqueousTM-Micro Total RNA Isolation Kit (Ambion, AM1931) following manufacturer’s instructions. At least 5 embryos were collected for each biological sample. Three distinct samples were analysed per condition. For single-end mRNA libraries, 2.5 ng of total RNA were used to amplify the cDNA using the SMART-Seq v4 Ultra Low Input RNA Kit (Clontech-Takara). 1 ng of amplified cDNA was used to generate barcoded libraries using the Nextera XT DNA library preparation kit (Illumina). Basically, cDNA is fragmented and adapters are added in a single reaction followed by an amplification and clean up. The size of the libraries was checked using the Agilent 2100 Bioanalyzer High Sensitivity DNA chip and their concentration was determined using the Qubit® fluorometer (ThermoFisher Scientific).

For single-end microRNA libraries, 500 pg of total RNA were used to generate barcoded miRNA-seq single-end libraries using the Bioo NEXTflex Small RNA (BiooScientific). Briefly, 3´ and 5´ SR adapters were first ligated to the RNA sample. Next, reverse transcription followed by PCR amplification was used to enrich cDNA fragments with adapters at both ends. Adapter-ligated cDNA fragments from different samples were pooled and run in a 6% polyacrilamide gel. The 147 nt band, corresponding to the pooled miRNA libraries, was purified from the gel. Finally, the quantity and quality of the pooled miRNA libraries were determined using the Agilent 2100 Bioanalyzer High Sensitivity DNA chip. Both, mRNA microRNA libraries were sequenced on a HiSeq 2500 (Illumina) and processed with RTA v1.18.66.3. FastQ files for each sample were obtained using bcl2fastq v2.20.0.422 software (Illumina).

For FastQC reads quality reports analysis, trimming of adaptors and alignment of sequences, fastq sequence reads were uploaded to the European version of the Galaxy platform [51]. The quality of the reads was analyzed with FastQC Read Quality reports (Galaxy Version 0.74 + galaxy1) software, trimmed with Trim Galore software (Galaxy Version 0.6.7 + galaxy0) and aligned to the built-in mouse reference genome mm10 (GRCm38) with the RNA STAR Gapped-read mapper (Galaxy Version 2.7.10b + galaxy3) [52].

For gene-expression analyses, bam files were downloaded from the Galaxy server and further analyzed with the different RStudio packages downloaded from the Bioconductor website (http://bioconductor.org, accessed on 2 March 2023). Reads were assigned to mRNA and microRNA genes by using the “featureCounts” function of the “Rsubread” package, version 2.10.5 [53]. In addition, mouse gencode.vM20.annotation.gff3 annotation file release M20, GRCm38.p6 (https://ftp.ebi.ac.uk/pub/databases/gencode/Gencode_mouse/release_M20/gencode.vM20.annotation.gff3.gz) and mmu.gff3 chromosomal coordinates of Mus musculus microRNAs miRBase v22 (https://www.mirbase.org/download/#:~:text=mml.gff3-,mmu.gff3,-osa.gff3) were used for mRNA and miRNA analysis, respectively. Uniquely mapped reads were used to calculate gene expression. The library size of each experimental point ranged from 18,625,406 to 26,021,292 sequences and from 601,840 to 1,251,790 sequences for mRNA and miRNA analysis, respectively. Fastq files and abundance measurements of features were uploaded to Gene Expression Omnibus database with GEO accession number: GSE189344.

Differential gene expression analysis was performed through package “edgeR-package” version 4.4.2 [54]. The normLibSizes() function was used to normalize the library sizes by trimmed mean of M-values (TMM) method. Only transcripts detected in three transcriptomes were used in the analysis. All gene comparisons with an adjusted p-value < 0.05 and an abslog2 fold change (FC) > 2 were considered differentially expressed under the experimental conditions. For miRNA-mRNA transcripts interaction analysis we used miRComb package [55]. Gene Set Enrichment Analysis (GSEA)-based Gene Ontology (GO) analyses were conducted with the “clusterProfiler” package version 3.6.0 [56, 57]. The gene sets with a p-value < 0.05 were considered overrepresented under the experimental conditions.

### RNA isolation and RT-qPCR

RNA samples from the same pools used for RNAseq libraries construction as well as additional isolated RNA samples were used; namely, those corresponding to E9.5 PE cells and E10.5 FACS sorted EE cells. All RT-qPCR experiments followed MIQE guidelines [58] and similarly as previously reported [59, 60]. Briefly, RNA from tissue samples was extracted and purified by using the Direct-zol™ RNA Miniprep kit (Zymo research) and the cell line RNA isolation was performed with ReliaPrep™ RNA Miniprep Systems kit (Promega), both according to manufacturer´s instructions. For mRNA/lncRNA expression measurements, 500ng of total RNA was used for retro-transcription with PrimeScript™ RT Master Mix (Takara), the resulting cDNA was diluted 1/5, both according to manufacturer’s guidelines. For microRNA expression analyses, 20 ng of total RNA was used for retro-transcription with with miRCURY LNA RT Kit (Qiagen), the resulting cDNA was diluted 1/40, following manufacturer´s guidelines. Negative controls, without reverse transcriptase, were performed for each sample to assess genomic contamination. Real-time PCR experiments were performed with 1 µL of cDNA, GoTaq qPCR Master Mix (Promega) and corresponding primer sets as described in Supplementary Table 1. All qPCRs were performed using a CFX384 TM thermocycler (Bio-Rad) following the manufacturer’s recommendations. For mRNA, the qPCR program consisted of 95 °C for 30 s (initial denaturalization), followed by 40 cycles of 95 °C for 5 s (denaturalization); 60 °C for 10 s (annealing); 75 °C for 7 s (extension). Finally, melting curves were determined by an initial step of 95 °C for 5 s, followed by 0.5 °C increments for 7 s from 65 °C to 95 °C. For microRNAs, the qPCR program consisted of 95 °C for 10 min (initial denaturalization), followed by 40 cycles of 95 °C for 5 s (denaturalization); 60 °C for 1 min (annealing and extension). Finally, melting curves were determined by an initial step of 95 °C for 5 s, followed by 0.5 °C increments for 7 s from 65 °C to 95 °C. The relative level of expression of each gene was calculated as described by Livak & Schmittgen (2001) [61] using *Gapdh* as the internal control for mRNA expression analyses and *5S* for microRNA expression analyses, respectively. Each PCR reaction was carried out in triplicate and repeated in at least three distinct biological samples to obtain representative means.

### Cell lines

In this study four cell lines were used, immortalized embryonic endocardial cell line MEVEC [62], muscle cardiac cell line HL1 (Sigma-Aldrich SCC065), mouse embryonic epicardial cell line MEC1 (Sigma-Aldrich SCC187) and epicardial cell line EPIC [63]. Each cell line was cultured following the manufacturer’s recommendations for 24 h at 37 °C in a humidified atmosphere of 5% CO_2_ at 4 × 10^4^ cells per well in plates of 24 wells before transfection.

### Tissue explants isolation

All experiments were performed with the approved consent of the Ethics Committee of the University of Jaén and Andalusian Regional Government (14/03/2022/038). Pregnant CD1 wild-type female mice were harvested to E10.5. E10.5 ventricles were manually dissected and cultured in DMEM/Glutamax culture medium. For embryonic epicardial cell isolation for qPCR analysis, the ventricles were dissected in Earle’s balanced salt solution (EBSS) (Gibco), and cultured in a 12-well plates with collagen type I gels (Sigma-Aldrich #C3867-1 VL), as previously described [59], for 48 h before transfection. Epicardial cells from transfected E10.5 ventricles were isolated, pooled and directly stored at −80°C until used. For confocal microscopy analyses, the ventricles were dissected in Earle’s balanced salt solution (EBSS) (Gibco), and cultured in a 4-chambered glass bottom dish with collagen treatment as previously described [59], 48 h before transfection. Briefly, samples were fixed in freshly made 4% PFA and stored in PBS at 4 °C until used. Each experimental condition was repeated at least three times with a minimum number of three explants per condition, respectively.

### microRNA mimics or anti-miR and siRNA transfections

E10.5 ventricles were cultured for 48 h at 37 °C in a cell culture incubator before administration of miRNAs mimics (pre-miRNAs), anti-miRNAs or siRNAs, respectively, as previously described [60]. Pre-miRNAs, anti-miRNA and siRNA transfections were carried out with Lipofectamine 2000 (Invitrogen), following the manufacturer’s guidelines. Briefly, 50nM of premiRNAs (microRNA precursor) or antimiRs (microRNA inhibitor) were applied to the explants [3 explants per well], and for siRNA transfection 60-80nM of siRNA were applied. These concentrations were selected based on preliminary experiments in which qRT-PCR was performed to assess transfection efficiency, adjusting the doses for each condition. After incubation, 24 h for pre-miR or 48 h for anti-miR and siRNA, explants were either processed for RT-qPCR or immunohistochemical (IHC) analyses. Negative controls, E10.5 ventricular explants, treated only with Lipofectamine were run in parallel. To perform IHC analyses, the explants were fixed in PFA 4% for 15 min at room temperature rinsed two times in PBS for 5 min and stored in PBS at 4 °C. For RT-qPCR analysis, explant epicardial outgrowths were collected and stored at −80°C.

### Cell migration assays

Mouse E10.5 ventricular explants were isolated from the developing embryo and the ventricular apex was dissected, plated upside down into coated collagen 4 chambered glass bottom dishes, incubated into DMEM Glutamax culture media for 48 h as previously reported. At this stage, emerging epicardial outgrowths start to develop. Transfections with corresponding pre-miRNAs, anti-miRNAs, scrambled, siRNAs and negative controls, respectively, were carried out and cultures were allowed to develop for another 24/48 h. Explants were rinsed in PBS for 5 min at room temperature and fixed in 4% PFA for 15 min at room temperature. After the fixation, the explants were rinsed two times in PBS for 5 min and incubated with Phalloidin FITC 1:1000 (Abcam) following the manufacturer’s recommendations. Finally, DAPI 1:2000 (Sigma) was incubated for 15 min at room temperature, rinsed two times in PBS for 5 min and stored in PBS in darkness at 4 °C. Subsequently, representative images of each explant were collected using a Leica TCS SP5 II confocal scanning laser microscope, and the extension of the epicardial migration (i.e. cohesive, non-cohesive and total migration) was measured in ten different regions per image, using ImageJ software. Mean and SD values were subsequently plotted.

### Confocal scanning laser microscopy analyses

Immunofluorescence analyses were performed as previously reported [59]. Briefly, control and experimental explants were collected after the corresponding treatment, rinsed in PBS for 10 min at room temperature, and fixed with 4% PFA at room temperature for 15 min. After fixation, the samples were rinsed three times [10 min each] in PBS at room temperature and then permeabilized with 0.02% Triton X-100, 50nM Nh_4_Cl and PBS for 10 min at room temperature. Non-specific binding sites were blocked with 0.2% Gelatin solution (Sigma-Aldrich) applied two times for 10 min. As primary antibodies, an anti-Wt1 (Santa Cruz) and anti-cTnT (Hytest) at 1:200 dilution in blocking solution was applied overnight at 4 °C. Ventricle explants were rinsed 3 times in PBS for 10 min and incubated with secondary antibody anti-Goat 488 (Invitrogen) 1:100 dilution, 30 min at room temperature. Finally, ventricle explants were incubated with DAPI 1:2000 (Sigma) for 15 min at room temperature and rinsed two times in PBS for 5 min each. The explants were stored in PBS in darkness at 4 °C until analysed using a Leica TCS SP5 II confocal scanning laser microscope.

### Statistical Analyses

For statistical analyses of datasets, unpaired Student’s t-tests were used. Significance levels or P values are stated in each corresponding figure legend. P < 0.05 was considered statistically significant.

## Results

### Coding and non-coding RNA differential expression in the PE to EE transition in mice

To investigate gene expression changes during the transition from PE to EE, we performed RNAseq on manually dissected PE from E9.5 Wt1-GFP heterozygous mouse embryos (n ~ 20) and GFP + FACS-sorted EE cells from E10.5 Wt1-GFP embryonic hearts (n ~ 15). Each condition included three independent biological samples (PE: n = 7–8 per sample; EE: n = 3–5 hearts per sample). RNAseq libraries for microRNAs and mRNA/lncRNAs in these two distinct stages of epicardial development were constructed and sequenced, yielding an average of 5*10^6^ reads (5,25*10^6^ ± 1*10^6^) for microRNA libraries and 35*10^6^ reads (36,5*10^6^ ± 2,5*10^6^) for mRNA/lncRNA libraries. Alignment efficiency was approximately 85–90% for mRNAs of the total input resulting in the identification of ~ 12,500 genes, while microRNA reads alignment yielded lower inputs, 40–50% of the total and identified ~ 200 microRNA expressed in both conditions. An exploratory analysis validated the similarity between PE E9.5 vs EE E10.5 RNAseq datasets (Supplementary Fig. 3).

**Fig. 1:**
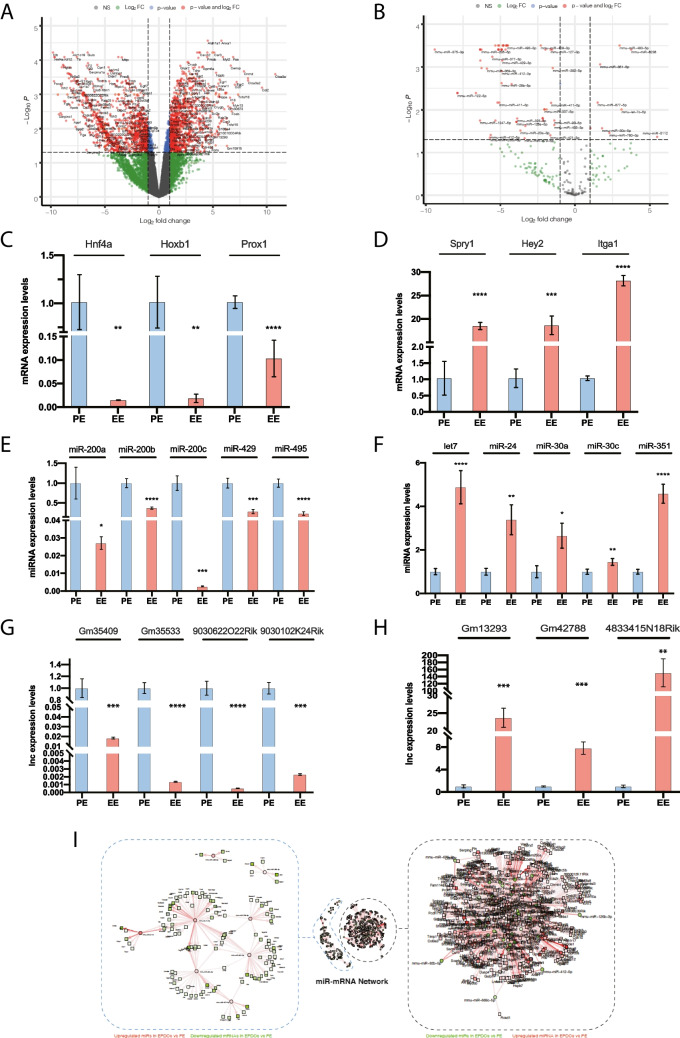
Panel **A**. Volcano plot of mRNA expression profile. Red dots represent significant DE genes in PE E9.5 *vs.* EE E10.5, right side downregulated mRNAs in PE9.5, left side upregulated mRNAs in PE9.5. Green dots represent mRNAs that were not DE genes in PE E9.5 *vs.* EE E10.5. Panel **B**. Volcano plot of miRNA expression profile. Red dots represent significant DE genes in PE E9.5 *vs.* EE E10.5, right side downregulated miRNAs in PE9.5, left side upregulated miRNAs in PE10.5. Green dots represent miRNAs that were not DE genes in PE E9.5 *vs.* EE E10.5. Panel **C**. RT-qPCR analysis for mRNA validation of DE genes in PE > EE (i.e. *Hnf4a*, *Hoxb1*, and *Prox1*) in PE9.5 and EE10.5 mouse tissues, demonstrating high expression in PE *vs.* EE (n = 3). Panel **D**. RT-qPCR analysis for mRNA validation of DE genes in PE < EE (i.e. *Spry1*, *Hey2*, and *Itga*) in PE9.5 and EE10.5 mouse tissues, demonstrating high expression in EE *vs.* PE (n = 3). Panel **E**. RT-qPCR analysis for miRNA validation of DE genes in PE > EE (i.e. *miR-200, miR-200b, miR-200c, miR-429* and *miR-495)* in PE9.5 and EE10.5 mouse tissues, demonstrating high expression in PE *vs.* EE (n = 3). Panel **F**. RT-qPCR analysis for miRNA validation of DE genes in PE < EE (i.e. *let-7c, miR-24, miR-30a, miR-30c* and *miR-351*) in PE9.5 and EE10.5 mouse tissues, demonstrating high expression in EE *vs.* PE (n = 3). Panel **G**. RT-qPCR analysis for lncRNA validation of DE genes in PE > EE (i.e. *Gm35409, Gm35533, 9030622O22Rik* and *9030102 K24Rik)* in PE9.5 and EE10.5 mouse tissues, demonstrating high expression in PE *vs.* EE (n = 3). Panel **H**. RT-qPCR analysis for lncRNA validation of DE genes in PE < EE (i.e. *Gm13293, Gm42788,* and *4833415 N18Rik*) in PE9.5 and EE10.5 mouse tissues, demonstrating high expression in EE *vs.* PE (n = 3). Panel **I**. Schematic representation of putative microRNAs-mRNAs interactions of DE genes mRNA in PE E9.5 *vs.* EE E10.5. *p < 0.05, **p < 0.01, ***p < 0.001, ****p < 0.0001

In order to identify those genes that might be involved in governing the transition between the PE and EE, we have identified those DE genes, including therein microRNAs, mRNAs and lncRNAs, using as selection criteria those genes displaying a log2 FC > 1 and FDR p < 0.05. This analysis identified 979 mRNAs up-regulated in the PE as compared to EE (Supplementary Table 1), whereas 886 display the opposite pattern, down-regulated in the PE as compared to the EE (Supplementary Table 2) (Fig. 1A). RT-qPCR validation confirmed the differential expression of these mRNAs (Fig. 1C-D). In this context, it is important to highlight that transcription factors such as *Hnf4a*, *Hoxb1* and *Prox1* are enriched in the PE at E9.5, whereas *Spry1*, *Hey2* and *Itga1* are enriched in the EE at E10.5.

Similarly, microRNA analysis identified 59 microRNAs highly expressed in PE as whereas 9 microRNAs were upregulated in EE (Supplementary Table 3) (Fig. 1B). RT-qPCR validation confirmed the differential expression of these miRNAs, where in fact, *miR-200a-3p, miR-200b-3p, miR-200c-3p, miR-429-3p* and *miR-495-3p* displayed higher level of expression in PE, whereas *let-7c-5p, miR-24-3p, miR-30a-3p, miR-30c-5p* and *miR-351-5p* showed higher expression levels in EE (Fig. 1E-F).

LncRNAs display an averaged lower expression levels as compared to protein-coding RNAs. Even so, our RNAseq analyses identified 60 lncRNAs that are highly expressed in the PE (Supplementary Table 4) and 111 lncRNAs upregulated in EE (Supplementary Table 5). Similar to mRNAs and miRNAs, the differential expression of some of these lncRNAs was confirmed by RT-qPCR, i.e. *Gm35409, Gm35533, 9030622O22Rik and 9030102 K24Rik* display high levels in PE and *Gm13293, Gm42788* and *4833415 N18Rik* display high levels in EE (Fig. 1G-H). These data demonstrate therefore an important contribution of miRNAs, mRNAs and lncRNAs in the PE to EE transition, yet their functional implications remain to be elucidated.

### Signaling pathway enrichment displays significant differences in mouse PE and EE differential expressed genes

To provide a comprehensive analysis of the biological processes associated with DE genes profile in PE and EE stages, we performed a Gene Set Enrichment Analysis (GSEA). As depicted in Supplementary Fig. 4 A, DE genes upregulated in EE display enhanced representation of myofilament and myosin complex, mitochondrial respiratory chain and actomyosin contractile and actin filaments in GSEA GO Cellular Compartment (CC) analyses. In contrast, DE genes downregulated in EE display enhanced representation of membrane and cell–cell contact pathways in GSEA GO CC analyses, suggesting a shift from intercellular communication in PE to muscle function and motility in EE. GSEA GO Molecular Function (MF) analysis further supported this distinction (Supplementary Fig. 4B). DE genes upregulated in EE display enhanced representation of chemokine receptor binding, tropomyosin and structural components of the muscle and muscle alpha-actinin binding, while those DE genes downregulated in EE display enhanced representation of cofactors and calcium ion binding and signaling receptor activity These data reinforce the role of DE genes in EE in muscle function and motility, whereas DE genes in PE remain more engaged with cell–cell signaling. Finally, the pathways revealed by GSEA GO Biological Pathway (BP) further support these findings (Supplementary Fig. 4C).

### Tissue-specific expression patterns of differentially expressed genes in PE and EE further support different signaling pathway enrichment

To further investigate the biological relevance of the DE genes between E9.5 PE and E10.5 EE, we analyzed their tissue-specific expression using the Genepaint database (https://gp3.mpg.de). Analyses of the top 10% downregulated DE genes (PE > EE) (~ 90 genes) revealed that approximately 55% (47/85) of them displayed restricted liver expression at E14.5 days, 9% (8/85) were preferentially expressed in the endocardium and 8% (7/85) display expression in the epicardium. No detectable expression was observed for 23% (20/85) of the DE genes analysed and 11% (10/85) were not found on the Genepaint database (Supplementary Fig. 5). Analyses of the top 10% downregulated DE genes (PE < EE) (~ 100 genes) revealed that approximately 17% (17/101) were expressed in the epicardium, 12% (12/101) in the endocardium and 6% (6/101) within the myocardium. No detectable expression was observed for 32% (32/101) of the DE genes analyzed and 17% (17/101) were not found on the Genepaint database (Supplementary Fig. 5). Overall, these data demonstrate a distinct bias on the preferential distribution of DE genes in the PE and EE stages. It is important to highlight in this context the large abundance of hepatic specific genes in the E9.5 PE fraction and a relatively low abundance of epicardial restricted genes. On the other hand, it is equally surprising that a small but consistent number of DE genes with enhanced expression in the EE 10.5 are mostly myocardial restricted.

### MicroRNA-mRNA regulatory networks reveal distinct transcriptional pathways involved in mouse PE to EE transition

Previous studies have identified microRNA-mRNA cross-talk correlations by searching for opposite patterns between mRNA and microRNAs in distinct experimental conditions [64–66]. Using miRComb software, we have searched for all putative candidate microRNAs that target each of the DE mRNAs with high expression in the PE as compared to the EE as depicted in Fig. 1I. Nine distinct microRNAs with enhanced expression in the EE (*let7c-5p, miR-351-5p, miR-30c-5p, miR-780-3p, miR-677-5p, miR-5112, miR-320-3p, miR-483-5p and miR-6236*) display complementary pattern to mRNAs with the opposite pattern (PE > EE). *Let7c-5p, miR-351-5p* and *miR-30c-5p* (*let-7c, miR-351* and *miR-30c*, respectively, will be used throughout this text) display a wide range of interactions, supporting a more relevant functional role, as compared to *miR-780-3p, miR-677-5p, miR-5112, miR-320-3p, miR-483-5p and miR-6236* (Fig. 1I, Supplementary Fig. 6). On the other hand, 60 distinct microRNAs with enhanced expression in the PE display a complementary pattern to mRNAs with the opposite pattern (PE < EE). *miR-495-5p, miR-200b-3p* and *miR-181c-5p* (*miR-495, miR-200b* and *miR-181c*, respectively, will be used throughout this text) are the three microRNAs that display a larger range of interactions, respectively, as compared to the other DE microRNAs (Fig. 1I, Supplementary Fig. 6). Overall, these data identify novel microRNA-mRNA predicted interactions that might be functionally important during PE/EE development.

We have centered our attention on those three DE microRNAs that display a larger number of mRNA interactions in each developmental stage, i.e. *let-7c, miR-351* and *miR-30c* in PE < EE and *miR-495, miR-200b* and *miR-181c* in PE > EE. Biological theme comparison of the molecular function of the putative DE-mRNA targets identified by these microRNAs revealed that *let-7c* is primarily involved in RNA polymerase/transcription factor DNA binding (Supplementary Fig. 7) while *miR-30c* is involved in receptor and cell–cell signaling as well as in ion channel regulation. *miR-495* is primarily involved in RNA polymerase, transcription factor DNA binding and GAG binding, while *miR-200b* in proteoglycan, GAG, calcium binding and GTPase receptor activity. Moreover, *miR-181c* is primarily involved in protein heterodimerization, nuclear receptor, transcription factor and steroid/hormone activity as well as on protein phosphatase and collagen binding. Similar findings are observed in CC and BP biological theme comparisons (data not shown). Importantly, there was a minimal overlap on the predicted targets between these key microRNAs, reinforcing the idea that they modulate different signaling pathways. *Let-7c, miR-351* and *miR-30c* display only one shared target (*Prtg*) (Supplementary Fig. 8A), while *miR-495, miR-200b* and *miR-181c* display equally uncommon shared targets (*Nfib, Mbnl2, Kat2b* and *Nr3c1*). However, an increased number of targets are shared between *miR-495* and *miR-200b* (12 genes; *Fn1, Rnd3, Elf2, Rapgef2, Plxna4, Gpm6a, Psd3, Amotl2, Arl4a, Hapln1, Dusp1, Vegfa*) and between *miR-495* and *miR-181c* (11 genes; *Col16a1, Acer3, Sox6, Dmxl2, Dusp6, Sept8, Gpr22, Akap6, Adamts5, Aqp4, Mcc*), suggesting common functional roles in signaling pathways (Supplementary Fig. 8B). Additionally, it worth mentioning that several shared DE mRNAs target of PE > EE miRNAs have a role modulating cell migration in other different biological context, i.e. Nfib [67–69], Mbnl2 [70], Nr3c1 [71, 72], Fn1 [73, 74] and Rnd3 [75, 76], a key process during PE/EE development.

### Differentially expressed microRNA-mRNA predicted interactions are distinctly validated in EPIC and MEC epicardial cells

**Fig. 2:**
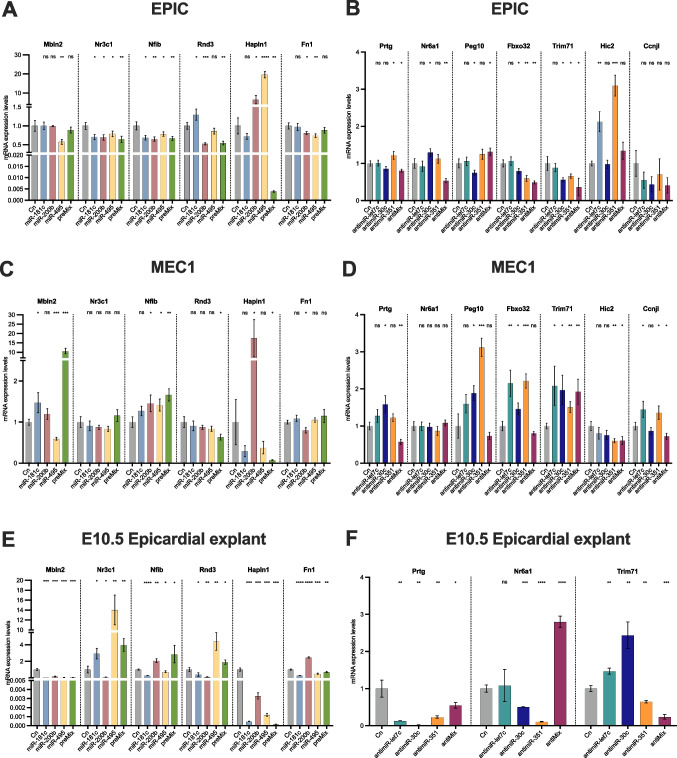
Panel **A**. RT-qPCR analysis for mRNA targets after microRNA gain-of-function (*miR-181c, miR-200b, miR-495* and *preMix*) in EPIC epicardial cells. Observe that selective mRNA downregulation is achieved after microRNA administration. Note that *Nr3c1* and *Nfib* are significantly decreased after all individual or combinatorial microRNA treatments (n = 3). Panel **B**. RT-qPCR analysis for mRNA targets after microRNA loss-of-function (*let-7c, miR-30c, miR-351* and *antiMix*) in EPIC epicardial cells. Selective mRNA upregulation is achieved for *Prtg*, *Nr6a1* and *Hic2* after individual microRNAs knockdown (n = 3). Panel **C.** RT-qPCR analysis for mRNA targets after microRNA gain-of-function (*miR-181c, miR-200b, miR-495* and *preMix*) in MEC1 epicardial cells. Observe that only *Mbln2* and *Hapln1* are downregulated after *miR-495* administration and Fn1 after *miR-200b* treatment (n = 3). Panel **D**. RT-qPCR analysis for mRNA targets after microRNA loss-of-function (*let-7c, miR-30c, miR-351* and *antiMix*) in MEC1 epicardial cells. Note that Fbxo32 and Trim71 are up-regulated after all individual microRNA inhibition (n = 3). Panel **E**. RT-qPCR analysis for mRNA targets after microRNA gain-of-function (*miR-181c, miR-200b, miR-495* and *preMix*) in EEx from E10.5 epicardial explants. Observe that *Mbln2* and *Hapln1* are decreased after all individual or combinatorial microRNA treatments. Note also that selective mRNA downregulation is achieved after microRNA administration for *Nr3c1*, *Nfib*, *Rnd3* and *Fn1* (n = 3). Panel** F**. RT-qPCR analysis for mRNA targets after microRNA loss-of-function (*let-7c, miR-30c, miR-351* and *antiMix*) in EEx from EE10.5 epicardial explants. Observe a selective up-regulation of Trim71 after let-7c and *miR-30c* inhibition (n = 3). *p < 0.05, **p < 0.01, ***p < 0.001, ****p < 0.0001

To dissect the regulatory mechanisms driven by DE-microRNAs, we performed gain-of-function assays of different microRNAs in two distinct epicardial cell lines, MEC1 and EPIC, respectively. MEC1 cells retain the morphology of early primary epithelial epicardial cells and express epicardium-specific markers including epicardin (*Tcf21*), *Tbx18* and *Krt18*, while EPIC cells continuously proliferate and expand, acquiring a characteristic mesenchymal phenotype and expressing mesenchymal markers such as *Sox9* [63, 77]. Overexpression of PE-enriched microRNAs *miR-181c, miR-200b* and *miR-495* in EPIC cells resulted in significant downregulation of predicted targets, including *miR-181c* reduced *Nr3c1*, *miR-200b* suppressed *Rnd3 and Fn1*, while *miR-495* decreased *Mbln2* and *Nfib* (Fig. 2A). In MEC1 cells, a similar pattern was observed for *Mbln2* downregulated by *miR-495* and *Fn1* by *miR-200b*, but *Nr3c1, Nfib* or *Rnd3* were not significantly affected (Fig. 2C). The combinatorial effect of these three microRNAs (preMix) led to a decreased expression of *Rnd3* and *Hapln1* in both epicardial cell lines, while *Nr3c1* and *Nfib* were downregulated exclusively in EPIC (Fig. 2A, C). To uncover if the functional role of microRNAs also occurs in other cardiovascular cell types, RT-qPCR analyses were also performed after DE microRNAs gain-of-function experiments in HL1 cardiomyocytes and MEVEC endocardial cells (Supplementary Fig. 9 A,B). Interestingly, our data demonstrated that neither *Mbln2* nor *Nfib* were downregulated in HL1 cardiomyocytes or MEVEC endocardial cells, while *Fn1* was impaired by *all the three microRNAs,* and *Nr3c1* and *Rnd3* were downregulated in HL1 cardiomyocytes. However, the preMix had a greater impact on target regulation in HL1 cardiomyocytes and MEVEC endocardial cells (Supplementary Fig. 9 A,B). In conclusion, it is worth highlighting that EPIC epicardial cells recapitulate most of the microRNA-mRNA target predicted interactions after miRNAs gain-of-function, except for *Hapln1*, supporting the notion that miRNAs PE > EE molecular regulation is cell type-specific.

In the same regard, loss-of-function assays were executed for EE-enriched microRNAs, *let-7c, miR-30c* and *miR-351*, in EPIC and MEC1 epicardial cells (Fig. 2B, D). Our RT-qPCR analysis led us to identify that in the EPIC cell line *miR-351* inhibition modulated the up-regulation of *Prtg*, whereas *Nr6a1* was up-regulated after the inhibition of *miR-30c* and *Hic2* after *let7c* or *miR-351* inhibition, respectively. On the other hand, neither *Peg10*, *Fbxo32, Trim71* nor *Ccnjl* expression were boosted after the loss-of-function of these microRNAs in EPIC cells (Fig. 2B). In the MEC1 cell line is relevant to point out that *Fbxo32* and *Trim71* were significantly up-regulated after *let-7c, miR-30c* and *miR-351* inhibition in MEC1 cell line (Fig. 2D), while *Peg10* was up-regulated after *miR-30c* and *miR-351* inhibition. Additionally, *Prtg* and *Ccnjl* were up-regulated after *miR-30c* and *let-7c* inhibition, respectively, in MEC1 cells. Curiously, *Nr6a1* and *Hic2* expression was not increased, in some cases (*miR-351*) even significantly decreased, after these microRNA inhibition (Fig. 2D). In this case is noteworthy that the combinatorial effect of these three microRNAs (antiMix) does not have any relevant effect on the up-regulation of the mentioned target genes except for *Trim71* in MEC1 and *Peg10* in EPIC cells (Fig. 2B, D). To summarize, our data demonstrate that MEC1 cells recapitulate most of the microRNA-mRNA target predicted interaction after miRNAs loss-of-function as compared to EPIC cells.

### Differentially expressed microRNA-mRNA predicted interactions are not recapitulated in ex vivo E10.5 epicardial outgrowths

To further dissect the regulatory mechanisms driven by DE-microRNAs, we performed gain- and loss-of-function assays of the PE > EE and PE < EE microRNAs, in mouse E10.5 epicardial explants. After 24 h of culture, Wt1^+^ epicardial cells (EEx) were transfected with microRNAs and collected after ventricular tissue removal (Supplementary Fig. 9 C). Gain-of-function experiments with PE > EE microRNAs *miR-181c, miR-200b, miR-495* demonstrated that epicardial expression of *Mbln2* and *Hapln1* was significantly decreased in all individual conditions, while *Nfib* and *Fn1* were negatively regulated after *miR-181c* and *miR-495* administration. Additionally, *Rnd3* was down-regulated by *miR-181c* and *miR-200b,* whereas *Nr3c1* down-regulation was specific to *miR-200b.* Finally, *Mbln2*, *Hapln1* and *Fn1* expression was significantly decreased after premix treatment (Fig. 2E). Comparing these findings with the previous in vitro experimental assays, EPIC cells recapitulated key interaction seen in EEx, such *miR-181c*-mediated *Nfib* suppression and *miR-200b*-driven regulation of *Nr3c1* and *Rnd3*. Finally *miR-495* controls *Mbln2, Nfib* and *Fn1*. However, discrepancies were observed, including *miR-181c* downregulating nearly all the predicted target genes except for *Nr3c1* ex vivo but not in vitro. Such discrepancies might be related to the myocardium-epicardium interaction ex vivo and/or the differential cell behaviour of in vitro epicardial cell lines vs E10.5 ex vivo epicardial explants.

Similarly, loss-of-function assays of the three PE < EE microRNAs, *let-7c, miR-30c* and *miR-351* in EEx revealed that *Trim71* expression was up-regulated after *let-7c* and *miR-30c* inhibition, in line with the observations in MEC1. However, *Prtg* and *Nr6a1* were down-regulated in all individual experimental conditions. The combinatorial effect of antiMix leads exclusively to *Nr6a1* up-regulation (Fig. 2F).

These results demonstrate intriguing discrepancies in the regulatory effects of DE microRNAs in both MEC1/EPIC cells and EEx from E10.5 explants, being needed to underscore the importance of considering cell-specific contexts in understanding the functional implications of the microRNAs in embryonic epicardial cells ex vivo.

### *MiR-495, let-7c,* and *miR-351* regulate epicardial cell migration

**Fig. 3:**
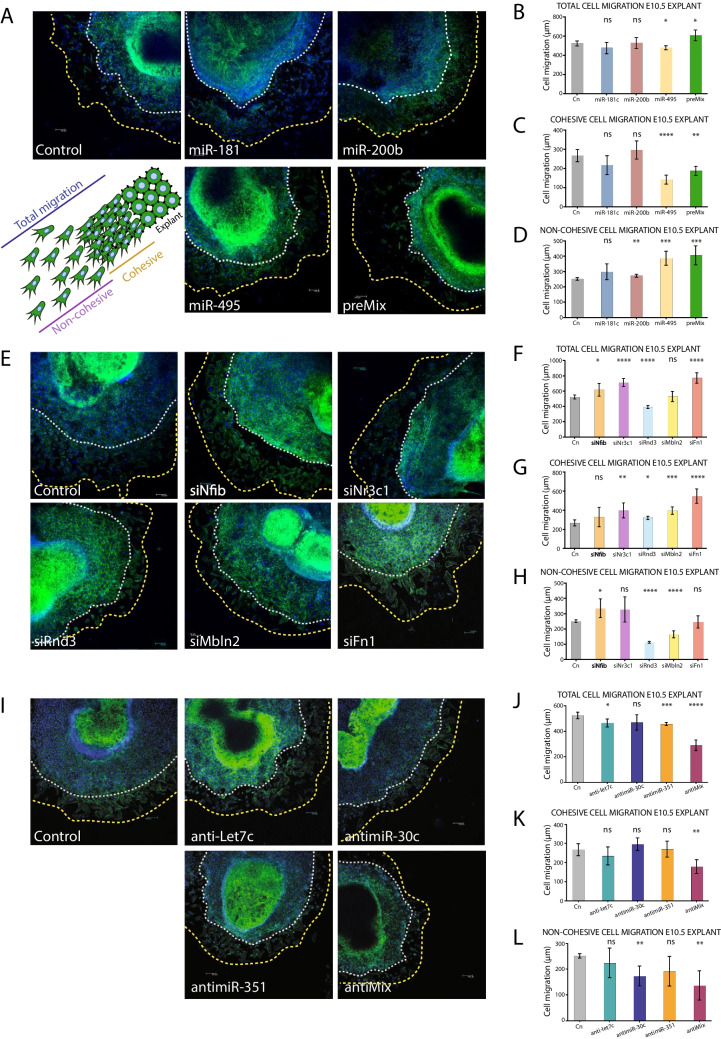
Panel A. Migration assays after DE microRNAs in PE > EE *miR-181c*, *miR-200b, miR-495* and *preMix* administration, respectively. Quantitative cell migration analysis guided by the schematic representation of cell migration quantification; *Total cell migration*, cellular migration from ventricular explant border to the outermost individual cell of the culture; *Cohesive cell migration*, collective migration from ventricular explant border; *Non-cohesive cell migration*, individual cell migration from the outermost periphery of the cohesive migration. Panel **B**, **C**, **D** Quantitative cell migration for DE microRNAs in PE > EE; *miR-181c*, *miR-200b, miR-495* and *preMix* administration*.* Note that *miR-495* administration impairs total cell migration followed by cohesive migration repression (n = 5). Panel** E**. Migration assays after DE mRNAs in PE < EE *Nfib, Nr3c1*, *Rnd3, Mbln2* and *Fn1* silencing. Panel **F**, **G**, **H** Quantitative cell migration for DE mRNAs in PE < EE; *Nfib, Nr3c1*, *Rnd3, Mbln2* and *Fn1* inhibition. Note that *Nr3c1* loss-of-function enhances total cell migration followed by cohesive migration increment (n = 5). Panel **I** Migration assays after DE microRNAs in PE < EE *let-7c, miR-30c, miR-351* and *antiMix* administration, respectively. Panel **J**, **K**, **L** Quantitative cell migration for DE microRNAs in PE < EE *let-7c, miR-30c, miR-351* and *Mix* inhibition*.* Note that *let-7c and miR-351* loss-of-function administration impair total cell migration without cohesive and non-cohesive migration affection (n = 5). *p < 0.05, **p < 0.01, ***p < 0.001, ****p < 0.0001

Epicardial cell migration is fundamental for heart development, as PE cells move by direct contact or through cell aggregates to cover the myocardium. This process begins at the atrioventricular canal region and, expands to form a continuous epithelial layer that eventually covers the entire heart. As the epicardium matures, EMT facilitates EPDC proliferation, migration, and differentiation, supporting coronary vasculature and ventricular growth. To assess the impact of microRNAs on this process, we performed gain- or loss-of-function assays in E10.5 ventricular explants and analyzed three different aspects. *Total cell migration*, representing all cellular migration from the explant ventricular border to the outermost individual cell of the culture; *Cohesive cell migration*, considering only those collective migrating cells from the explant ventricular border; and finally, *non-cohesive cell migration*, representing only those individual migrating cells from the outermost periphery of the cohesive migration (Fig. 3A). Our data demonstrated that total cell migration was impaired after *miR-495* overexpression and enhanced after preMix treatment, while no significant outgrowth differences were noted for *miR-181c* and *miR-200b* experimental conditions. Cohesive migration was repressed after *miR-495* and preMix treatment while non-cohesive migration was enhanced after *miR-200b* and *miR-495* gain-of-function, as well as, preMix treatment (Fig. 3A-D). In this regard, we observed that epicardial cells within the non-cohesive outgrowth exhibit a reduced size compared to controls following miR-495 gain-of-function, with a concurrent increase in Myh9 protein expression in MEC1 epicardial cells in vitro, whereas no differences are observed in F-actin polymerization (Supplementary Fig. 10 A-B). Thus, these data demonstrate that differential expression of distinct microRNAs can selectively modulate total, cohesive and non-cohesive epicardial cell migration.

As previously mentioned, our RT-qPCR analysis unveiled a specific explant epicardial cells regulatory miRNA-mRNA crosstalk (Fig. 2E). To further elucidate the role of the PE < EE DE-mRNAs in epicardial cell migration, E10.5 cardiac explants were transfected with *Mbln2*, *Nfib*, *Nr3c1*, *Rnd3* and *Fn1* siRNAs. Our epicardial cell migration analysis evidenced that total cell migration was enhanced after *Nfib, Nr3c1* and *Fn1* inhibition and decreased after *siRnd3* treatment, while no significant outgrowth differences were noted for *siMbln2*. Cohesive migration was promoted after loss-of-function of all mRNAs, except for *Nfib*, while non-cohesive migration was enhanced only by *Nfib* loss-of-function, and decreased after *Rnd3* and *Mbln2* inhibition, whereas *Nr3c1* and *Fn1* did not exerted any effect on non-cohesive cell migration (Fig. 3E-H). Thus, our data demonstrate that DE-mRNAs in PE *vs.* EE can selectively modulate total, cohesive and non-cohesive epicardial cell migration.

A similar analysis was performed for those PE < EE DE-microRNAs, where cell migration was assessed through loss-of-function experiments targeting individual microRNAs *let-7c*, *miR-30c*, *miR-351* and antiMix. Our results demonstrated that total cell migration was decreased after *antilet-7c, antimiR-351* and antiMix treatment, while no significant outgrowth differences were observed after *antimiR-30c* administration. Cohesive migration was repressed only after antiMix treatment without significant effect mediated by individual conditions, and finally, non-cohesive migration was impaired by *miR-30c* loss-of-function, as well as, antiMix treatment (Fig. 3I-L). These results align with our findings that epicardial cells in the non-cohesive outgrowth do not exhibit a reduced area compared to controls following *let-7c* loss-of-function, and no significant difference in Myh9 protein expression is observed in MEC1 epicardial cells in vitro, although F-actin polymerization is impaired (Supplementary Fig. 10 A-C). Therefore, these data further underscore the functional role of DE-microRNAs in epicardial cell migration.

Importantly, it should be highlighted that *miR-495, let-7c* and *miR-351* exert significant effects on epicardial cell migration, underscoring the importance of their precise regulation for proper epicardium formation and EPDCs migration.

### *MiR-181c, miR-200b,* and *let-7c* administration promotes cardiomyogenic cell specification in vitro but not ex vivo

**Fig. 4:**
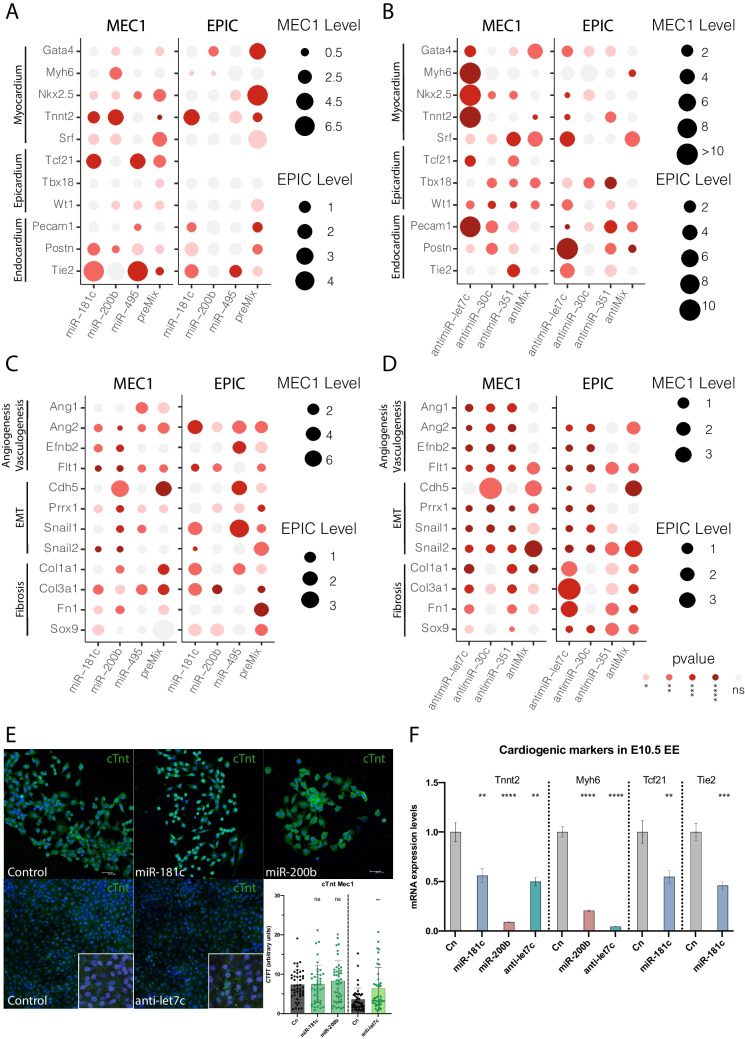
Panel **A**, **B** Analysis of lineage specific markers expression, i.e. myocardial (*Gata4, Myh6, Nkx2.5, Tnnt2, Srf*), epicardial (*Tcf21, Tbx18, Wt1*), and endocardial (*Pecam1, Postn, Tie2*) after *miR-181c*, *miR-200b, miR-495* and *preMix* administration or *let-7c, miR-30c, miR-351* and anti*Mix* inhibition, respectively (n = 3). Panel** C**, **D** Analysis of biological processes markers, i.e. angiogenesis (*Ang1, Ang2, Efnb2, Flt1*), EMT (*Cdh5, Snail1, Snail2*), and fibrosis (*Col1a1, Col3a1, Fn1, Sox9*) in MEC1 and EPIC epicardial cells after *miR-181c*, *miR-200b, miR-495* and *preMix* administration or *let-7c, miR-30c, miR-351* and anti*Mix* inhibition, respectively (n = 3). Panel **E**. Representative images of immunohistochemical analyses of cardiac troponinT (cTnt) in MEC1 epicardial cells, after administration of *premiR-181c, premiR-200b*, and *anti-let7c* as compared to controls. Observe that there is a significant difference in the expression of cTnt after *let-7c* inhibition (n = 5). Panel **F**. RT-qPCR analyses of selected cardiogenic markers (Tnnt2, Myh6, Tcf21, Tie2) in EEx from E10.5 epicardial explants after *premiR-181c, premiR-200b*, and *anti-let7c* administration (n = 5). *p < 0.05, **p < 0.01, ***p < 0.001, ****p < 0.0001

As outlined previously, after the embryonic epicardium covers the naked myocardium, it subsequently undergoes EMT leading to EPDCs that thereafter differentiate into distinct cell types such as cardiac fibroblasts, vascular smooth muscle cells, pericytes, endothelial and fat cells and possibly, also into cardiomyocytes. To dissect the role of DE microRNAs during cell lineage specification, epicardial cells (EPIC and MEC1) were overexpressed with *miR-181c, miR-200b, miR-495* and preMix, respectively (Fig. 4A, C, and Supplementary Fig. 11). Linage specific markers of epicardium (*Wt1, Tcf21* and *Tbx18*), myocardium (*Gata4, Nkx2.5, Srf, Tnnt2* and *Myh6*) and endocardium (*Pecam1, Tie2* and *Postn* as an endocardial derivative marker) as well as markers for EMT (*Cdh5, Snail1, Snail* and *Prrx1*), fibrogenesis (*Col1a1, Col3a1, Fn1* and *Sox9*) and angio-vasculogenesis (*Ang1, Ang2, Efnb2* and *Flt1*) were analysed by RT-qPCR. *miR-181c* gain-of-function did not evidence enhancement of early myocardial markers (i.e. *Gata4, Nkx2.5*), except for *Srf* in MEC1, but upregulated the cardiomyocyte terminal differentiation marker *Tnnt2*, in both epicardial cell lines. Moreover, endocardial and epicardial lineage markers were slightly increased such as *Tie2*, and *Postn* in epicardial MEC1 and EPIC cells and *Tcf21* only in epicardial MEC1 cells (Fig. 4A). In the same line, *miR-495* overexpression promoted the expression of endocardial lineage specification markers as revealed by upregulation of *Tie2* in both epicardial cell lines and *Postn* in MEC1 cells. Additionally, *miR-495* promotes the expression of the epicardial marker *Tcf21* in MEC1 as well as the cardiomyocyte terminal differentiation marker *Tnnt2* in EPIC. Finally, *miR-200b* did not exert any modulation on endocardial, myocardial and epicardial markers in EPIC cells, nonetheless the overexpression of *miR-200b* in MEC1 leads to an increment of myocardial markers such as *Srf, Myh6* and *Tnnt2* (Fig. 4A). When the three microRNAs are overexpressed together, it can be observed an increment of myocardial markers, for instance, early cardiogenic transcription factor as *Nkx2.5* is upregulated in both epicardial cells similarly as *Srf*, whereas *Gata4* is increased only in EPIC. Finally, epicardial markers as *Tcf21* and *Wt1* are upregulated in MEC1 and EPIC epicardial cells, respectively (Fig. 4A). Therefore, these data demonstrate that DE microRNAs in PE > EE can distinctly modulate epicardial-derived lineage specification.

Given the role of the epicardium during cardiogenesis, in order to have further insight of the molecular mechanisms driven by the DE microRNAs, we analysed by RT-qPCR, the expression levels of different molecular markers related with EMT, fibrogenesis and angiogenesis after gain-of-function experiments. For EMT markers all three DE-microRNAs exerted a marked repression of *Cdh5, Snail1, Snail2,* and *Prrx1* in MEC1, except for *miR-200b* which only promoted *Cdh5* gene expression. However, in EPIC cells, the gain-of-function of the *miR-181c* promoted the expression of *Snail1*, and *miR-495* induced *Snail1* and *Cdh5* expression, while *miR-200b* did not exert any significant effect over EMT markers (Fig. 4C). For angio-vasculogenesis markers analysis, no significant effect was observed after microRNA gain-of-function except for angiopoietins, i.e. *Ang1* in MEC1, mediated by *miR-495* and Ang2 in EPIC, regulated by *miR-181c* and *miR-495*. Notwithstanding, *miR-181c* and *miR-495* overexpression slightly promoted the expression of fibrogenic markers such as *Col1a1* and *Col3a1* in EPIC and MEC1 epicardial cell lines, respectively (Fig. 4C). Finally, EMT, angio-vasculogenesis and fibrotic molecular markers were upregulated by combinatorial miRNA overexpression, i.e.: *Ang2, Efnb2, Snail2, Fn1* and *Sox9* in EPIC epicardial cells, and *Ang1, Ang2, Cdh5, Col1a1, Col3a1* and *Fn1* in MEC1 (Fig. 4C). In sum, these results support the notion that *miR-181c* and *miR-495*, but not *miR-200b*, could modulate cell lineage specification promoting epicardial, endocardial and fibrogenic markers, whereas *miR-181c* and *miR-200b* promote myocardial markers expression in MEC1 epicardial cells (Supplementary Fig. 11).

Similar to DE-miRNAs in PE > EE, *let7c, miR-30c,* and *miR-351* loss-of-function experiments were performed in epicardial EPIC and MEC1 cells. Specific cardiac cell lineage and biological processes markers were studied (Fig. 4B,D, Supplementary Fig. 12). In this analysis, we observed that *anti-let7c* consistently promoted upregulation of early cardiogenic lineage markers in both epicardial cell lines, such as *Gata4* and *Srf,* whereas *Nkx2.5* and terminal differentiation markers such as *Myh6* and *Tnnt2* were only upregulated in MEC1 (Fig. 4B). In addition, the epicardial cell lineage markers are slightly promoted in EPIC cells through increment of *Tbx18* and *Wt1* expression as well as *Tcf21* in MEC1. Finally, endocardial markers revealed that loss-of-function of *let-7c* induced endocardial cell specification in EPIC cell line endorsed by *Post1* and *Tie2* upregulation.

In the same scenario of loss-of-function, *miR-30c* and *miR-351* modulated myocardial and epicardial markers in EPIC leading to the upregulation of *Gata4, Nkx2.5* and *Tnnt2*, as well as *Tbx18*, however, the modulation of these miRNAs in MEC1 cells did not have any impact on cardiogenic markers except for *Srf* expression. Finally, we evidenced that *miR-351* inhibition has an impact on the promotion of endocardial linage specification markers in both epicardial cells endorsed by *Pecam1, Postn* and *Tie2* expression, and the loss-of-function of *miR-30c* modulates *Pecam1* and *Postn* in MEC1 epicardial cells (Fig. 4B).

To get further insights into the functional role of the DE-miRNAs PE < EE other biological processes such as, EMT, fibrogenesis and angio-vasculogenesis representative markers were analysed in a loss-of-function model for *let-7c, miR-30c* and *miR-351,* respectively. Our RT-qPCR data demonstrated that all of them were significantly downregulated in MEC1 epicardial cells (Fig. 4D). However, loss-of-function of DE-miRNAs, *let-7c* and *miR-351* in EPIC cells, promoted the expression fibrogenic markers such as *Col1a1, Col3a1* and *Fn1*. Moreover, *antimiR-351* but no *anti-let7c* nor *antimiR-30c* promoted the expression of angio-vasculogenic markers as *Flt-1*. Finally, the antiMix treatment in both epicardial cells modulate EMT markers by the upregulation of *Snail1* in MEC1 and *Snail2* in MEC1 and EPIC cells (Fig. 4D). In sum, these results support the notion that *let-7c* can modulate cell lineage specification promoting myocardial markers while *miR-351* promotes endocardial lineage specification in epicardial cells. Moreover, these two miRNAs have a marked effect on the modulation of cardiac fibrogenic markers in EPIC epicardial cells (Supplementary Fig. 12). Furthermore, *let-7c* loss-of-function but no *miR-181c* and *miR-200b* gain-of-function, increases Tnnt2 protein expression levels as observed in MEC1 epicardial cells (Fig. 4E).

Since we have previously demonstrated the dynamic modulation of the lineage specification markers in epicardial cell lines by these microRNAs, we sought to investigate if the expression of epicardial, myocardial and/or endocardial markers were induced ex vivo in EEx. For this purpose, gain- and loss-of function of *miR-181c*, *miR-200b* and *let-7c* were performed in mouse E10.5 epicardial explants. RT-qPCR analysis evidenced that overexpression of *miR-181c* did not promote myocardial, endocardial or epicardial markers ex vivo. Moreover, *miR-200b* overexpression repressed *Tnnt2* and *Mhy6* expression in mouse EEx from E10.5 epicardial explants and similar effects were observed after *let-7c* inhibition ex vivo (Fig. 4F). Thus, these data suggest that the function of these three microRNAs in the embryonic epicardial cell specification is limited, underscoring the potential influence of other molecular factors as well as the neighbouring myocardial and endocardial tissues.

### Differentially expressed microRNAs cross-talk modulates epicardial cell specification

**Fig. 5:**
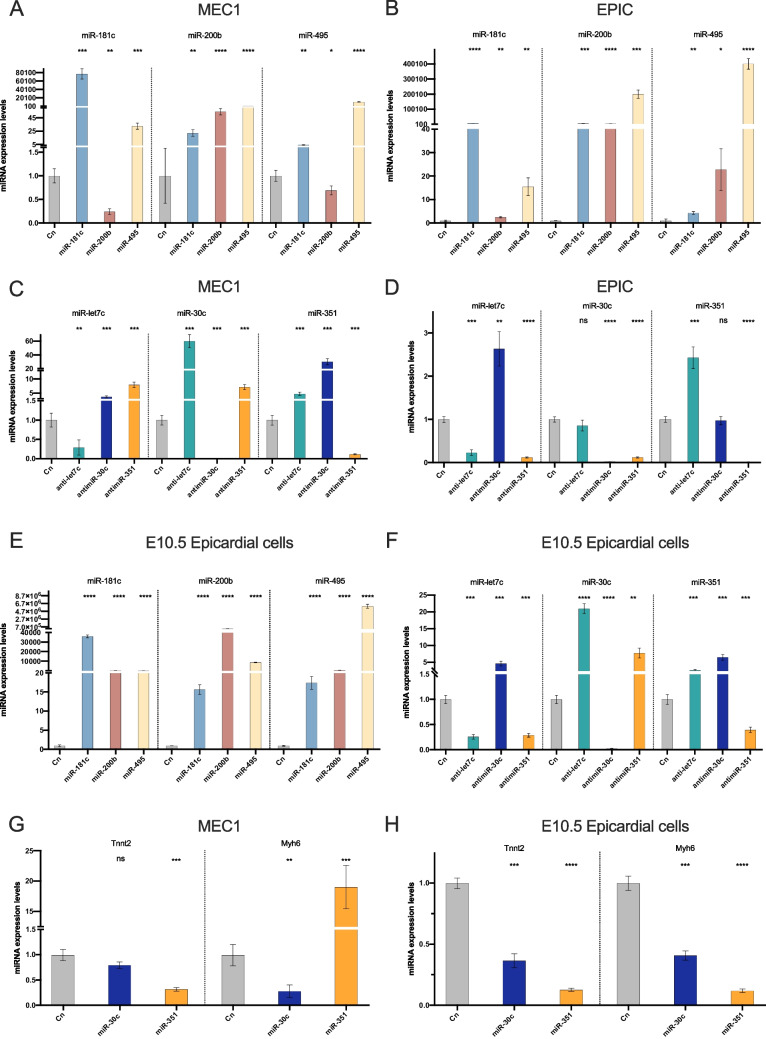
Panels **A**,** B**,** E** RT-qPCR analysis for microRNA expression after microRNA gain-of-function (*miR-181c, miR-200b, miR-495*) (n = 3). Panel **A** MEC1 epicardial cells. Panel** B** EPIC epicardial cells. Panel** E** EEx from E10.5 epicardial explants. Observe that microRNA upregulation is achieved after *miR-181c* and *miR-495* administration in all epicardial cell types and for *miR-200b* only in E10.5 epicardial cells. Panels** C**,** D**,** F** RT-qPCR analysis for microRNA expression after microRNA loss-of-function (*let-7c, miR-30c, miR-351*) (n = 3). Panel** C** MEC1 epicardial cells. Panel** D** EPIC epicardial cells. Panel **F** EEx from E10.5 epicardial explants. Observe that let-7c inhibition leads to high expression levels of *miR-30c* and *miR-351* in both epicardial cell types. Panel **G**, **H** RT-qPCR analysis of myocardial lineage markers (*Myh6, Tnnt2)* in MEC1 and EEx from E10.5 epicardial cells after *miR-30c* and *miR-351* gain-of-function. Observe that *miR-351* promotes *Myh6* expression only in MEC1 epicardial cells (n = 5). *p < 0.05, **p < 0.01, ***p < 0.001, ****p < 0.0001

Despite our comprehensive understanding of the fundamental principles underlying miRNA biogenesis and function, novel and unexpected aspects within these processes underscore the complexity of miRNA regulation. To get further insight of the functional regulation of the DE microRNAs, we conducted a RT-qPCR analysis of their mutual molecular regulation. In this regard, we analysed the expression levels of each microRNA following individual microRNA gain- or loss-of-function in MEC1 and EPIC epicardial cells as well as in embryonic epicardial cells (Fig. 5A-F). As expected, our results evidenced that *miR-181c, miR-200b* and *miR-495* administration were upregulated following miRNA gain-of-function in all epicardial cells, respectively (Fig. 5A, B, E). Moreover, overexpression of *miR-181c* enhanced *miR-200b* and *miR-495* expression and similarly, *miR-495* gain-of-function promoted *miR-181c* and *miR-200b* expression in epicardial cells (Fig. 5A, B, E). Notably, overexpression of *miR-200b* led to a decreased expression of *miR-181c* and miR-495 in MEC1 but not in EPIC or EEx (Fig. 5A, B, E). Similarly, analyses of loss-of-function assays also demonstrated a downregulation of *let-7c*, *miR-30c* and *miR-351* in all epicardial cells, as expected (Fig. 5C, D, F). However, loss-of-function experiments targeting *let-7c* resulted in elevated expression of *miR-30c* in MEC1 but not in EPIC epicardial cells, alongside increased *miR-351* expression in both cell types, a trend also observed in EEx (Fig. 5C, D, F). Comparable results were noted upon *miR-30c* inhibition, with upregulation observed in *let-7c* in MEC1, EPIC, and EEx, and *miR-351* only in MEC1 and EEx. Finally, after *antimiR-351* treatment, *let-7c* expression in MEC1 and *miR-30c* expression in MEC1 and EEx were elevated (Fig. 5C, D, F). Therefore, these data demonstrate a microRNA cross-talk regulation between differentially expressed microRNAs, a process that is also cell-type specific. To further analyse microRNA cross-talk, we performed in vitro experiments blocking transcription by using α-amanitin and we found that *miR-30c* and *miR-351* were upregulated when *let-7c* is inhibited, indicating that this microRNA acts by enhancing these two microRNAs at post-transcriptional levels (data not shown).

Since we have previously observed that the inhibition of *let-7c* displays an increment of *miR-30c* and *miR-351* expression in MEC1 and EEx, we sought to investigate the molecular implications of this regulatory feedback in cardiomyogenic cell lineage markers. *miR-30c* and *miR-351* gain-of-function experiments were conducted in MEC1 and EEx (Fig. 5G, H). Our results evidenced that *Tnnt2* expression levels are not increased following modulation of *miR-30c* nor *miR-351* in both MEC1 and EEx, whereas *Myh6* expression levels were upregulated specifically after *miR-351* overexpression in MEC1 epicardial cells (Fig. 5G, H). Thus, the cardiogenic role exerted by the inhibition of *let-7c* is not mediated by the modulation of these microRNAs, as only *miR-351* modulates *Myh6* expression.

Overall, our comprehensive analysis demonstrates that gain- or loss-of-function of one microRNA had a notable effect on the expression of other microRNAs, evidencing the intricate interplay between DE-microRNAs in regulating epicardial cell behaviour and highlighting their potential functional role in cardiac development.

### *Foxf1* modulates epicardial cell specification into myocardial and endothelial lineages via *let7c* and *miR-30c* regulation in vitro

**Fig. 6:**
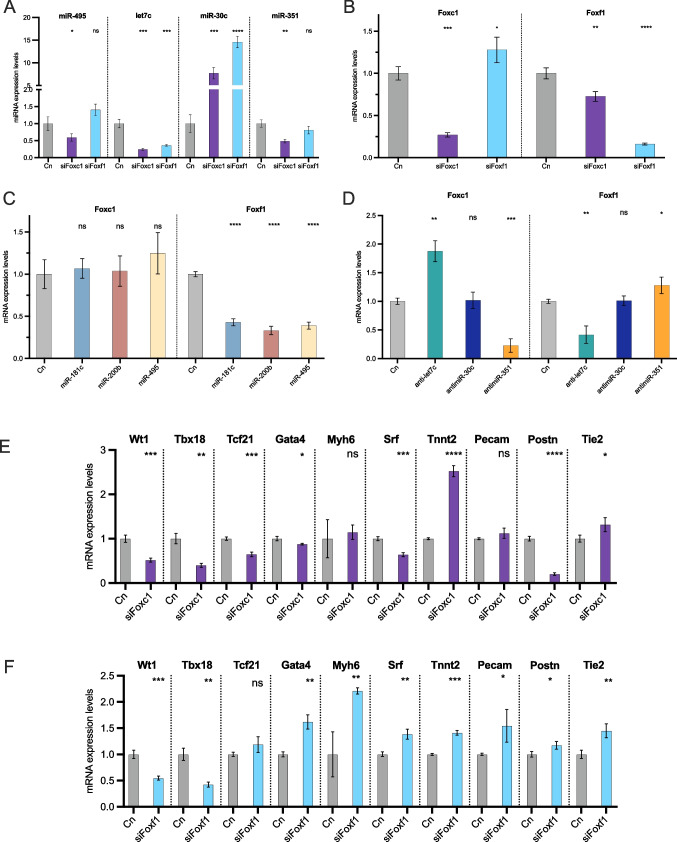
Panel **A**. RT-qPCR analysis of DE microRNAs (*miR-495, let-7c, miR-30c, miR-351*) after *Foxc1* and *Foxf1* siRNA treatment in MEC1 epicardial cells. Observe a differential microRNA regulation. Note also a significant downregulation of *let-7* and upregulation of *miR-30c* after *Foxf1* inhibition (n = 3). Panel** B**. RT-qPCR analysis of *Foxc1* and *Foxf1* expression after *Foxc1* and *Foxf1* siRNA treatment in MEC1 epicardial cells, respectively (n = 3). Panel **C**. RT-qPCR analysis of *Foxc1* and *Foxf1* expression after DE microRNAs in PE > EE treatment (*miR-181c, miR-200b, miR-495*) in MEC1 epicardial cells. Observe that *Foxf1* is downregulated after pre-miRNA administration (n = 3). Panel **D**. RT-qPCR analysis of *Foxc1* and *Foxf1* expression after DE microRNAs in PE < EE treatment (*let-7c, miR-30c, miR-351*) in MEC1 epicardial cells. Note that *Foxf1* is downregulated only after let-7c inhibition (n = 3). Panel** E**, **F**. RT-qPCR expression analysis of cardiogenic markers; epicardial (*Wt1, Tbx18, Tcf21*), myocardial (*Gata4, Myh6, Srf, Tnnt2*) and endocardial (*Pecam1, Postn, Tie2*) in MEC1 epicardial cells after *Foxc1* and *Foxf1* siRNA treatment, respectively. Observe that selective regulation is achieved after *Foxc1* and *Foxf1* inhibition. Note also that myocardial and endocardial lineage markers are upregulated after *Foxf1* inhibition (n = 3). *p < 0.05, **p < 0.01, ***p < 0.001, ****p < 0.0001

As we have previously mentioned, several mRNAs were significantly differentially expressed in PE (E9.5) and EE (E10.5). Among them, we have observed that there are 47 transcription factors (~ 4.6% of total DE mRNAs) with enhanced expression in PE, i.e., *Tbx5, Sox18, Prox1*, *Foxf1* (Supplementary Table 1), whereas 22 (~ 2.4%) display the opposite pattern, down-regulated in the PE as compared to the EE, e.g. *Tbx18, Sox9, Lhx9, Foxc1* (2**)**. FOX (Forkhead box) proteins are a family of transcription factors that play important roles in regulating gene expression that govern cardiogenesis. Basal expression analysis for *Foxc1* and *Foxf1* in EPIC, MEC1, MEVEC, HL1 and EEx revealed that *Foxc1* is abundantly expressed in EEx compared with the similar expression evidenced in EPIC and MEC1, while *Foxf1* is significantly downregulated in EEx and EPIC compared with MEC1 (Supplementary Fig. 13 A). Since *Foxf1* is expressed in the PE and *Foxc1* in EE we investigated whether these DE transcription factors, i.e. *Foxc1* and *Foxf1*, could modulate the expression of our DE-microRNAs in MEC1 epicardial cells. *Foxc1* inhibition resulted in decreased *miR-495* and *miR-351* expression, while loss-of-function of both *Foxc1* and *Foxf1* led to reduced *let-7c* and increased *miR-30c* expression (Fig. 6A). We subsequently examined whether these DE transcription factors exhibit also cross-talk regulation, similar as the DE microRNAs. Inhibition of *Foxc1* in MEC1 cells resulted in decreased expression of the *Foxf1* transcription factor, while inhibition of *Foxf1* in MEC1 cells led to upregulation of *Foxc1* expression (Fig. 6B**)**. To further elucidate the intricate regulatory network involving our DE-miRNAs, we assessed the expression levels of *Foxc1* and *Foxf1* in MEC1 epicardial cells overexpressed with the DE-miRNAs in PE > EE. No significant differences in expression levels were observed for *Foxc1* after *miR-181c*, *miR-200b* and *miR-495* administration while *Foxf1* expression was decreased after premiRs treatment (Fig. 6C). Conversely, inhibition of *let-7c* consistently upregulated *Foxc1* expression, exerting the opposite effect over *Foxf1*. Similarly, loss-of-function of *miR-351* resulted in decreased expression of *Foxc1* and increased expression of *Foxf1*. However, inhibition of *miR-30c* did not yield significant differences (Fig. 6D). Finally, we aimed to elucidate the involvement of the DE-transcription factors in epicardial cell lineage specification and migration. We analysed the expression levels of epicardial, myocardial and endocardial markers following loss-of-function of *Foxc1* and *Foxf1* in MEC1 epicardial cells. Our findings revealed that inhibition of *Foxc1* represses epicardial lineage specification markers such as *Wt1, Tbx18* and *Tcf21*, similar to those observed after *Foxf1* inhibition except for *Tcf21* that displayed no significant differences (Fig. 6E, F). Moreover, loss-of-function of *Foxc1* resulted in increased expression levels of *Tnnt2* and *Tie2*, while *Foxf1* inhibition promoted the expression of myocardial markers such as *Gata4, Myh6, Srf* and *Tnnt2*, as well as endocardial markers such as *Pecam1, Tie2 and Postn*. (Fig. 6E, F). Finally, epicardial cell migration in MEC1 following *Foxf1* loss-of-function lead to similar results as those observed for *let7c* inhibition in vitro, with no significant difference in Myh9 protein expression, although F-actin polymerization was impaired (Supplementary Fig. 13B).

In conclusion, our study unveiled complex regulatory networks orchestrated by DE-microRNAs and DE-transcription factors. These findings offer valuable insights into the regulatory mechanisms governing epicardial cell specification and cardiac development, particularly mediated by *Foxf1, let-7c* and *miR-30c*.

## Discussion

In this study, we provide novel insights into the dynamic regulation of microRNAs and their functional role in proepicardial (PE) and embryonic epicardial (EE) formation in mice. Our findings reveal distinct microRNA expression patterns that selectively influence key cellular processes, including cell–cell signaling, migration, and lineage specification during epicardial development. Specifically, we demonstrate that Foxf1 modulates the expression of *miR-495*, *miR-351*, and *let-7c*, which in turn regulate epicardial cell migration and myocardial specification. Notably, our results suggest a previously unrecognized microRNA-microRNA regulatory network, shedding light on the intricate molecular crosstalk driving epicardial development. These discoveries provide new mechanistic evidence of the transcriptional and post-transcriptional regulation orchestrating PE and EE formation, with potential implications for cardiac regenerative strategies.

microRNAs are short non-coding RNAs with tissue-specific expression that exert regulatory roles over different cellular processes ranging from embryonic development to pathological response [64–66]. In the cardiovascular system, several laboratories including ours, have yielded substantial evidence elucidating the microRNA differential expression during cardiogenesis [44–47, 78]. Moreover, *Dueñas *et al*.* (2020) recently evidenced a microRNA differential expression during PE and EE formation in chicken [48], however, the functional role of microRNAs in PE and EE development in mice and their application to enhance cardiogenesis remains elusive. In this study, we provide evidence about the microRNA differential expression pattern during PE and EE formation and their functional implications in mice. We found a large subset of microRNAs and mRNAs that display increasing expression in PE *vs*. EE, suggesting a plausible role in cell–cell signaling during proepicardial cell specification, differentiation and vesicles formation for direct contact and attachment of the proepicardial cells to naked myocardium in mice [5, 8, 38, 79–83]. On the other hand, a small subset of microRNAs displays increased expression, as well as high mRNA expression levels in EE *vs.* PE, supporting a modulatory role in the coordination of epicardial-derived signals involved in muscle function, coronary development, as well as myocardial growth [84–87]. Thus, these data revealed that differential microRNA signatures can selectively influence different signaling pathways during PE and EE formation.

The PE derives from the LPM and is formed at the venous pole of the heart during embryonic development in E9.5 mice. Lumenized vesicles from the proepicardial surface attach to the naked myocardium subsequently forming the embryonic epicardium at E10.5 [5, 8, 88]. Epithelial cells undergo EMT forming EPDCs that migrate into the myocardium, proliferate and differentiate into different cell types, including coronary endothelial cells, smooth muscle cells and cardiac fibroblasts, whereas their contribution to cardiomyocytes remain controversial [16, 19, 23–25, 50, 89–98]. Several laboratories have identified microRNA-mRNA cross-talk correlation during cardiac development (see recent reviews; [99, 100]). Brønnum et al. [101] have elucidated that *miR-21* modulates epicardium development and EPDCs fate-decision, through the established interplay between *Pdcd4* and *Spry1*, whereas Pontemezzo et al. [85] reported that *miR-200c* after Tfg-ß administration impact on the epithelial-to-mesenchymal transition process in epicardial cells. Our RNAseq analysis evidenced a complementary expression pattern of microRNAs and putative mRNA targets in PE *vs*. EE. Those microRNAs with enhanced expression in PE such as, *miR-495-5p, miR-200b-3p* and *miR-181c-5p,* have a plausible role blocking mRNA target expression (i.e. *Mbln2, Nr3c1, Nfib, Rnd3, Hapln1,* and *Fn1*) during the morphogenetic induction of proepicardial to embryonic epicardial cells transition. Herein, we demonstrate that *miR-495* regulates *Mbln2* in epicardial cells but not in myocardial nor endocardial cells, providing novel mechanistic insight into the role of microRNAs in this context. In addition, microRNAs upregulated in EE, such as *let7c-5p, miR-351-5p,* and *miR-30c-5p*, are repressing inductive signals derived from mRNAs targets (i.e. *Prtg, Nr6a1, Peg10, Fbxo32, Trim71, Hic2* and *Ccnjl*) during EMT and epicardial cell specification process, illustrated by the fact that *let-7c* regulates *Trim71* in epicardial cells. In summary, these findings further support the notion that tight regulation of microRNA-mRNA interaction plays a crucial role in coordinating cellular processes, i.e. cellular migration and lineage specification during PE and EE formation [4, 10, 23, 27, 34, 35, 95, 102–107]. Moreover, our results highlight the dynamic rewiring of miRNA-mRNA interaction during cardiogenesis, which modulate mRNA target expression in a cell-specific manner in epicardium, myocardium and endocardium. These differences may arise from variation in target mRNA expression, the presence of RNA-binding proteins that modulate microRNA function, or competing endogenous RNAs, as previously reported for microRNAs in other biological contexts [108–110]. These findings underscore the complexity of miRNA-mediated gene regulation in epicardial, reflecting the intricate nature of post-transcriptional networks across different cellular context during cardiac development. The discrepancies between individual miRNAs and the combinatorial conditions likely stem from interaction within broader miRNA networks producing non-linear outcomes. Additionally, cell-specific factors in MEC1 and EPIC cells contribute to these differences, emphasizing the dynamic regulation of gene expression during cardiac development.

microRNAs can modulate multiple biological processes, including cell migration in homeostatic and pathological conditions [111–115]. Cell migration is a key biological process during PE formation, such PE cells initially migrate onto the heart to establish the embryonic epicardium primarily at ventricular level, subsequently across the atrial chamber. Following EMT, EPDCs migrate into the myocardium to support coronary vasculature and ventricular development [116]. We provide herein evidences that *let-7c* and *miR-351* in the EE have an important role in controlling epicardial cell migration, in line with previous reports in other biological contexts [117–119]. Additionally, *miR-495* modulates epicardial cell migration, partially mediated by *Nr3c1,* in line with recent reports in pathological conditions [71, 72, 120–123]. Similarly, we evidenced that *Rnd3* is involved in epicardial cell migration, although is not coordinated with *miR-181c* and *miR-200b* modulation [75, 76]. These findings underscore the functional role of microRNAs and mRNAs in regulating epicardial cell migration, a highly relevant process in PE and EE formation, through the differential expression of cytoskeletal proteins. Given that defective migration of PE and EE cells can lead to severe congenital heart defects by disrupting coronary vasculature, myocardial growth, EMT and valve formation, our findings emphasize the pivotal role of miRNA-mRNA interactions in ensuring the precise regulation of epicardial cell migration, which is essential for proper cardiac morphogenesis.

As extensively documented, the epicardium plays a key role during cardiogenesis since a subset of EPDCs undergo EMT, migrate into the myocardium and differentiate into different cardiovascular lineages, i.e. coronary vascular smooth muscle cells, cardiac fibroblasts, endothelial cells, contributing to complete heart formation. Furthermore, it has been suggested that epicardial progenitors can also contribute to the cardiomyocyte lineage, although this statement remains controversial [50, 93, 97, 98]. In our study, we explored the plausible contribution of microRNAs in the process of epicardial cell lineage specification. Our findings indicate that the administration of *miR-181c* and *miR-200b,* as well as the inhibition of *let-7c* facilitates epicardial cell specification into the myocardial cell lineage in vitro but these effects were not observed ex vivo in the explant model. This discrepancy arises from the fundamental differences between the simplified in vitro environment and the more complex, physiologically relevant conditions of the explant model. Hence, these data support the notion that *miR-181c*, *miR-200b* and *let-7c* modulate myocardial specification of embryonic epicardial cells, underscoring the potential impact of molecular regulation induced by the neighbouring myocardial and endocardial tissues. Additionally, the induction of fibrotic markers in the epicardial cells observed after loss-of-function of *let-7c* suggests that this microRNA plays a broader role in modulating epicardial cell fate. This finding implies that *let-7c* may influence fibrotic processes during cardiac development, consistent with its established roles in regulating fibrosis in other biological contexts [124, 125].

During embryogenesis, extracellular information is needed for cells to make decisions during development and differentiation [126, 127]. Tight cross-talk between different signaling pathways such as TGF-β/BMP, Wnt/Wg, Hedgehog (Hh), Notch, and mitogen-activated protein kinases (MAPK) have been thoroughly described [128–133]. Similarly, cross-talk between transcription factors has been evidenced, e.g. Gata4-Tbx5 controls cardiac septum formation [134] and Tbx5-Nkx2.5 interaction promotes cardiomyocyte differentiation [135]. Nevertheless, there is scarce evidence regarding the plausible microRNA cross-talk that could modulate the maturation and expression of other microRNAs [136]. Our analysis demonstrated that DE microRNAs in PE and EE regulate the expression of other microRNAs in epicardial cells at post-transcriptional levels. Therefore, this is, to the best of our knowledge the first evidence that microRNAs can regulate the expression of other microRNAs, supporting thus functional implications for PE and EE development. Given the role of *let-7c* in terminal myocardial differentiation and its role in the regulation of *miR-30c* and *miR-351* expression, we evidenced that only *miR-351* promotes myocardial terminal differentiation in epicardial cells, promoting *Myh6* expression. These findings elucidate that in spite of the intricate interplay among DE microRNAs in PE *vs*. EE, governing epicardial cell behaviour, the terminal myocardial differentiation exerted by *let-7c* is not solely mediated by the EE > PE microRNAs interplay.

**Fig. 7:**
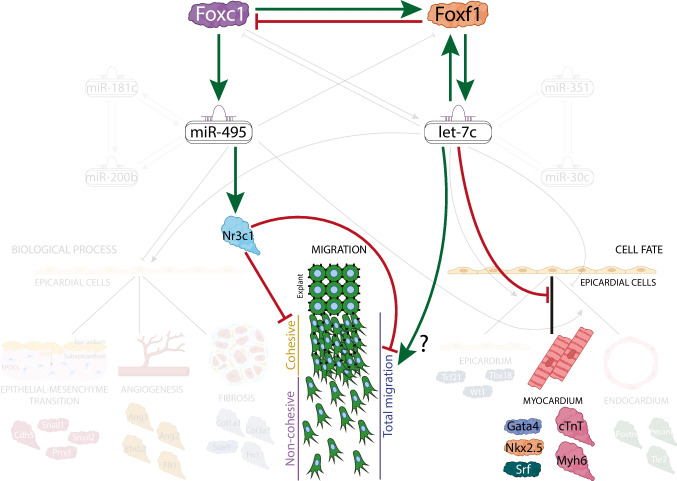
Schematic representation of the functional role of *Foxf1*, *miR-495* and *let-7c* in epicardial cell migration and specification. The figure illustrates the intricated molecular network regulating epicardial cell migration and specification, highlighting the molecular interplay between the transcription factors *Foxf1* and *Foxc1*, as well as their regulatory effect on *miR-495* and *let-7c*. This intricate molecular interplay manifests functionally in both epicardial cell migration and epicardial cell specification into cardiogenic lineage as highlighted in color. Additionally, in the background, the diagram also reflects the molecular crosstalk among distinct microRNAs and the role of *miR495* and *let-7c* in epicardial cell lineage specification into epicardium and endocardium, along with their involvement in other biological processes such as EMT, angiogenesis and fibrosis (Grey). Pointed arrows denote positive regulation, blunt arrows negative regulation and dashed lines no significant regulation

Cardiac-specific transcription factors such as *Nkx2.5* [137–139], *Mef2c* [140–142], *Pitx2* [143–146], *Srf* [147, 148] and *Fox* [149–153], are fundamental in both cardiogenesis and the development of PE and EE [37, 38, 154, 155]. Moreover, *Tbx18* is highly expressed in PE and essential in epicardium and coronary vasculature development [33, 104]. *Tcf21* and *Tbx5* are essential for mature proepicardial cells to establish contact with the myocardium and properly form the epicardium [35, 156]. *Wt1* is crucial for EMT of epicardial cells [28]. These transcription factors exert transcriptional control over multiple downstream targets, including both coding and non-coding RNAs and miRNAs, particularly those pivotal for heart development. In our RNAseq analysis, *Foxf1* displays enhanced expression in PE, whereas *Foxc1* shows an opposite expression pattern, with high expression in EE. Our data analysis revealed that *Foxc1* and *Foxf1* exert transcriptional control over the DE microRNAs in PE and EE during development, in line with previous report demonstrating similar transcription factor-miRNA transcriptional regulation i.e. *Pitx2*-miRNAs in a skeletal-muscle context [60]. In PE and EE formation and specification, it is worth highlighting that *Foxf1* controls *let-7c* and *miR-30c* expression. In addition, DE microRNAs in PE *vs.* EE, i.e. *miR-495* and *let-7c* modulate *Foxc1* and *Foxf1* expression and similar to those observed effects for these microRNAs, both transcription factors exhibit a cross-talk modulation. Overall these findings reveal a complex transcription factor *vs*. microRNA regulation in PE and EE formation and specification in epicardial cells during cardiogenesis (Fig. 7).

Understanding of the molecular mechanisms driving PE and EE formation and specification has greatly advanced over the last decade including the functional role of microRNAs (see recent reviews [157–159]). Regarding the tight molecular regulation of transcription factors and DE microRNAs in PE *vs.* EE, and the previously observed implication of microRNAs during epicardial cell specification, we evidenced that *Foxc1* modulates the expression of myocardial and endocardial markers (i.e. *Tnnt2* and *Tie2*), whereas *Foxf1* controls early cardiogenesis (i.e. *Gata4* and *Srf*), as well as cardiomyocyte terminal differentiation (i.e. *Tnnt2* and *Myh6*). Therefore, these observation supports the notion that transcription factors *Foxc1* and *Foxf1* modulate EE specification, being essential during cardiogenesis as previously reported [149–153, 160–164]. Therefore, mechanistically this study evidenced that *Foxf1* controls epicardial cell specification towards cardiomyocytes by modulating *let-7c* (Fig. 7).

In summary, we provide herein evidence that PE and EE formation and specification are biological processes tightly regulated by DE microRNAs and mRNAs during cardiogenesis. We demonstrated that *Foxf1* transcription factor modulates *miR-495, miR-351 and let-7c* expression and these microRNAs regulate epicardial cell migration and myocardial specification, hinting the essential co-regulatory role of transcription factor *vs.* microRNA for cardiogenesis during embryonic development (Fig. 7).

Despite the valuable insights provided by this study, some limitations should be acknowledged. While our RNA sequencing data suggest potential microRNA-mRNA regulatory interactions, further exploration to fully elucidate the regulatory networks that govern cardiogenesis would strengthen our findings. Additionally, future studies employing genetic loss- and gain-of-function models in mice would be necessary to confirm the role of specific microRNAs in PE and EE development. Lastly, although our study suggests potential translational applications for cardiac regenerative medicine, additional research is needed to determine whether modulating these microRNA pathways could enhance epicardial cell contribution to cardiac repair in postnatal or adult hearts.

## Conclusions

Our study highlights the intricate regulatory mechanisms orchestrated by differentially expressed microRNAs and mRNAs during the formation and specification of the PE and EE in cardiogenesis. We provide novel evidence that specific microRNAs, such as *miR-495* and *let-7c*, play crucial roles in modulating epicardial cell migration and myocardial specification. The transcription factor *Foxf1* regulates *let-7c* expression, thereby promoting key developmental processes as myocardial lineage specification from epicardial cells. Our findings underscore the complexity and importance of the microRNA-mRNA interaction networks and their co-regulatory roles with transcription factors in governing cardiogenesis. This study advances our understanding of the molecular mechanisms underlying heart development and highlights potential therapeutic targets.

## Supplementary Information

Below is the link to the electronic supplementary material.

Supplementary file1 (PDF 1.77 MB)

Supplementary file2 (PDF 639 KB)

Supplementary file3 (PDF 57.4 KB)

Supplementary file4 (PDF 2.13 MB)

Supplementary file5 (PDF 1.86 MB)

Supplementary file6 (PDF 32.7 KB)

Supplementary file7 (PDF 96.4 KB)

Supplementary file8 (PDF 523 KB)

Supplementary file9 (PDF 2.25 MB)

Supplementary file10 (PDF 7.08 MB)

Supplementary file11 (PDF 430 KB)

Supplementary file12 (PDF 438 KB)

Supplementary file13 (PDF 4.01 MB)

Supplementary file14 (XLSX 202 KB)

## Data Availability

RNAseq data were uploaded into Gene Expresssion Onmibus platform with accession number GSE189344. https://www.ncbi.nlm.nih.gov/geo/query/acc.cgi?acc=GSE189344

## Abbreviations

Anti-miRNA: MicroRNA inhibitor
AV: Atrioventricular
cDNA: Complementary DNA
DE: Differentially expressed
Dapi: 4',6-Diamidine-2'-phenylindole dihydrochloride
E8.5: Embryonic day 8.5
E9.5: Embryonic day 8.5
E10.5: Embryonic day 10.5
EBSS: Earle’s balance salt solution
EE: Embryonic epicardium
EEx: Wt1^+^ epicardial cells
EMT: Epithelial to mesenchymal transition
EPDCs: Epicardial derived cells
FBS: Fetal bovine serum
FC: Fold change
GEO: Gene Expression Omnibus
GO: Gene ontology
GSEA: Gene Set Enrichment Analysis
HEPES: 4-(2-Hydroxyethyl)- 1-piperazineethanesulfonic acid
IHC: Immunohistochemical analyses
lncRNA: Long non-coding RNA
LPM: Lateral plate mesenchyme
PBS: Phosphate buffer saline
PE: Proepicardium
Pre-miRNAs: MicroRNA precursor
RT-qPCR: Reverse transcriptase-quantitative polymerase chain reaction
SV: Sinus venosus
TMM: Trimmed mean of M-values
