# Supplementary figures and images for: Foxf1-mediated co-regulation of miR-495 and let-7c modulates epicardial cell migration and myocardial specification
Source: Cell Mol Life Sci. 2025 Jun 25;82(1):254. doi: 10.1007/s00018-025-05735-4 (PMC12187632; doi:10.1007/s00018-025-05735-4)

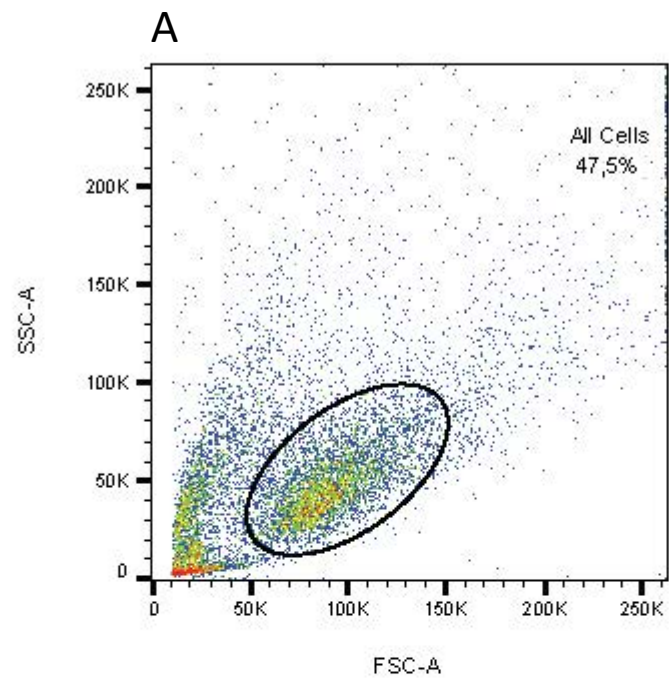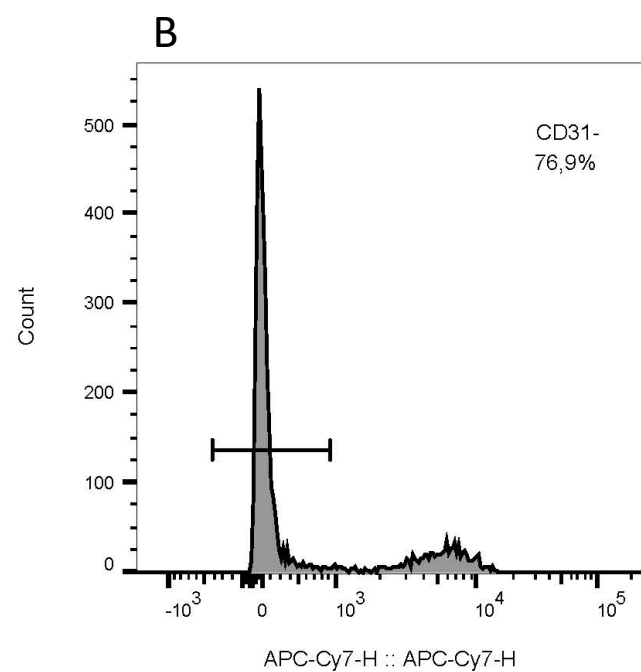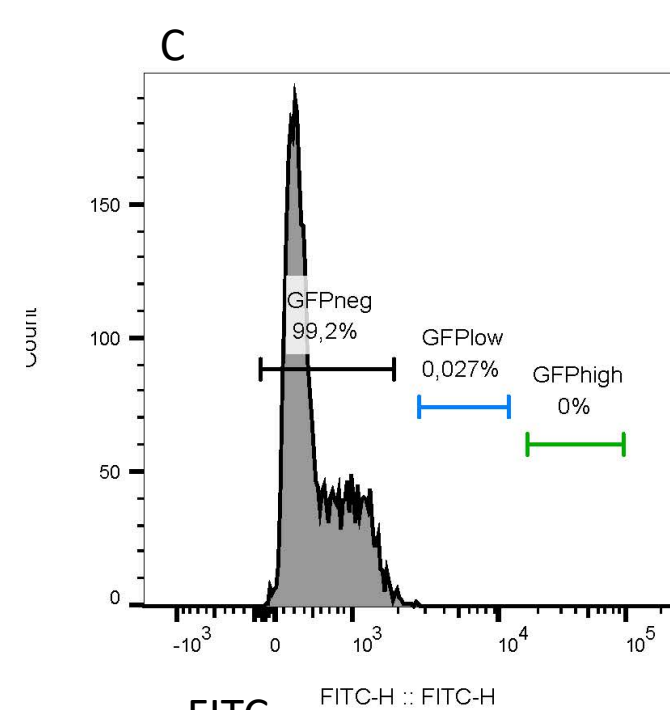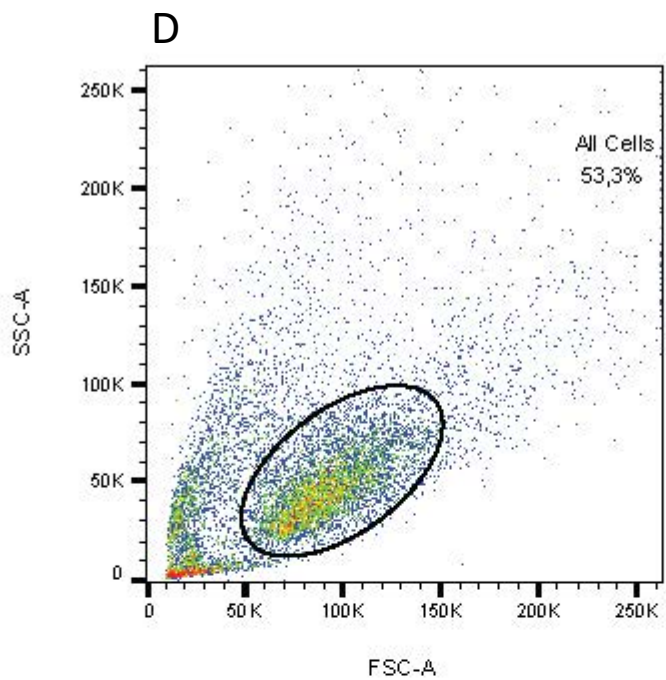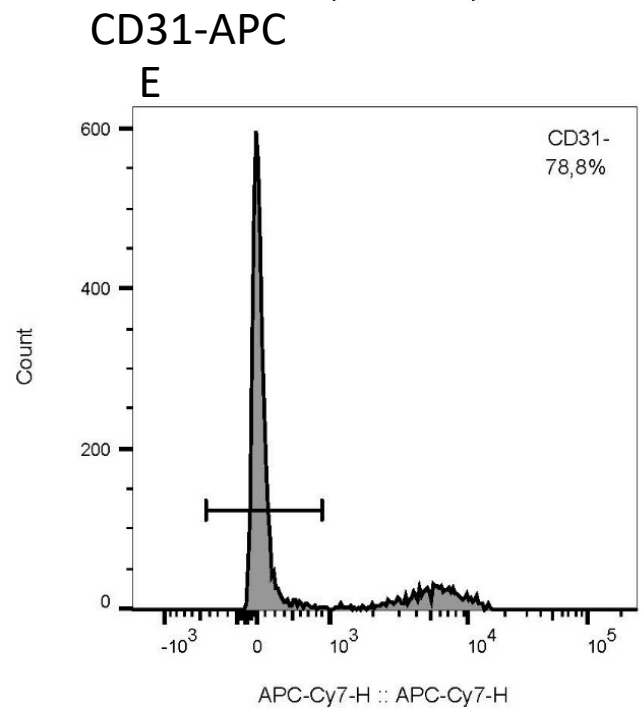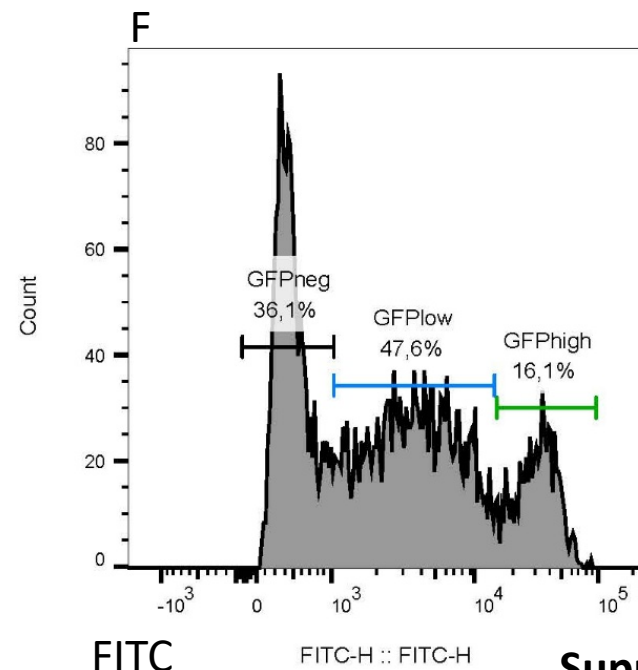

**Supplementary Figure 2**

Supplement: Supplementary file 2 — Supplementary file2 (PDF 639 KB) [file 18_2025_5735_MOESM2_ESM.pdf]

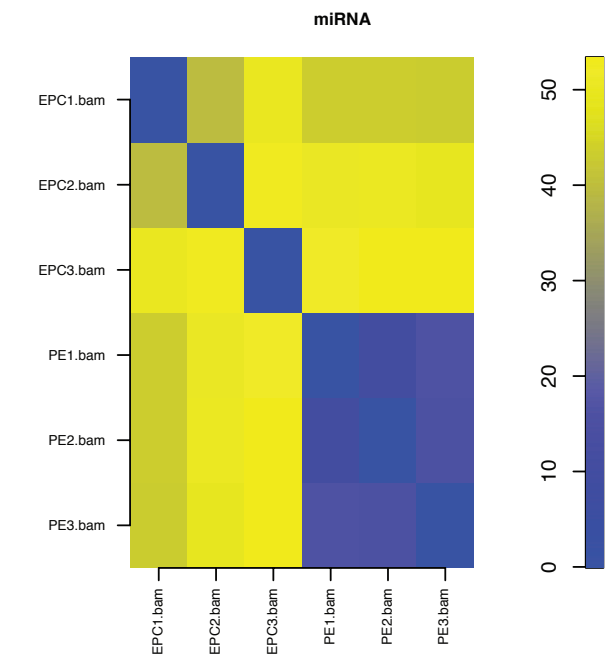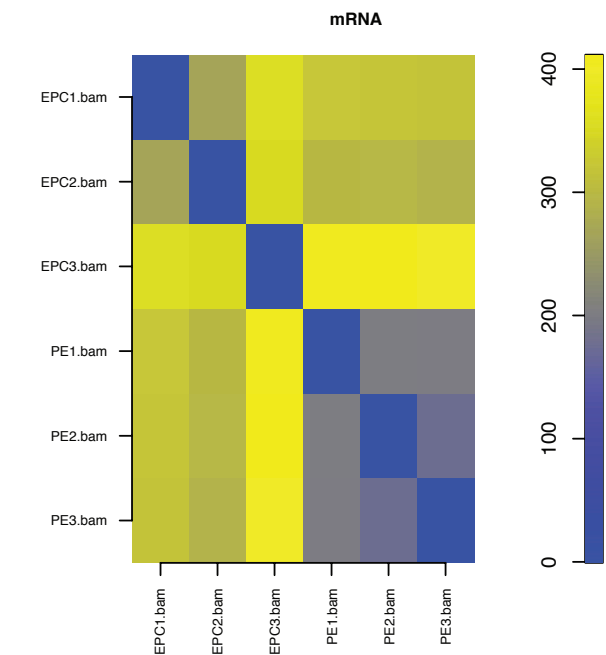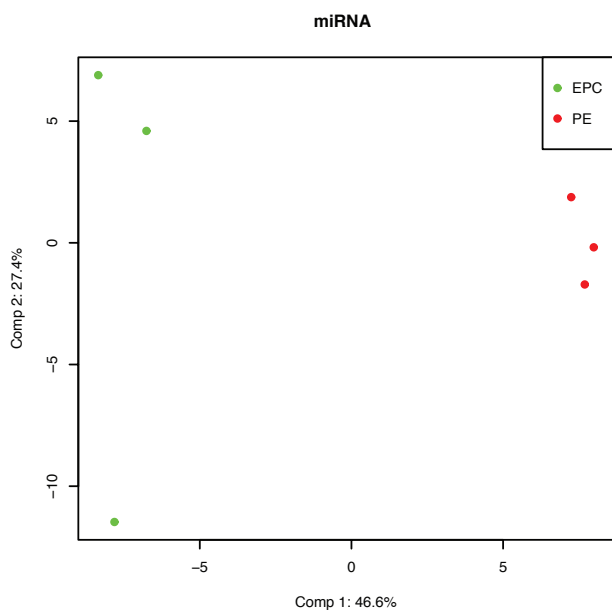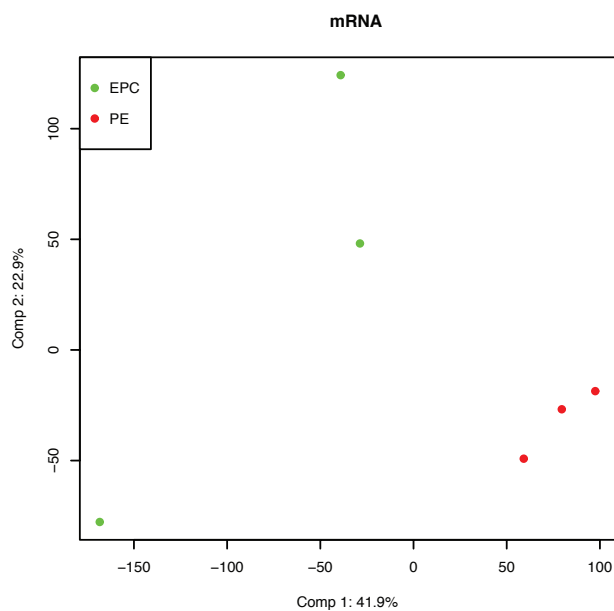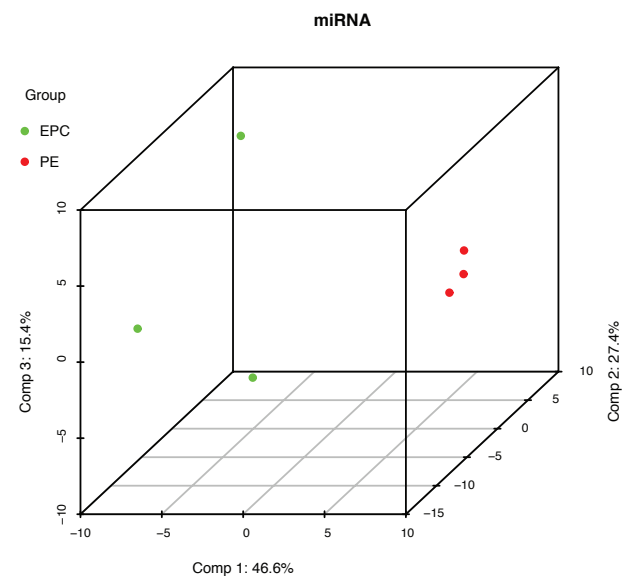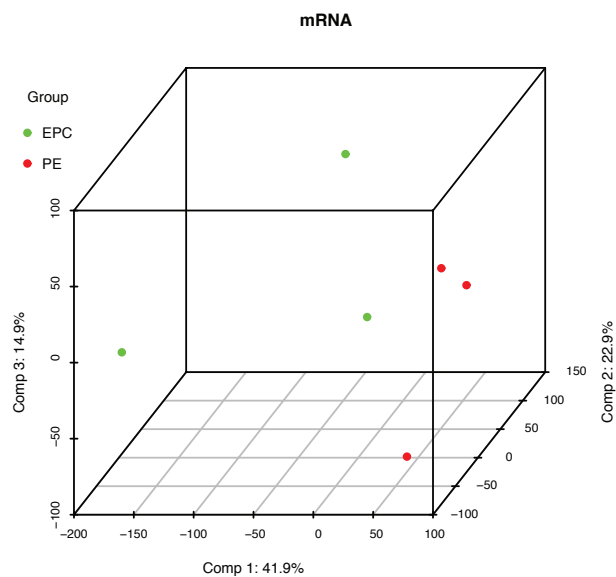

**Supplementary Figure 3**

Supplement: Supplementary file 3 — Supplementary file3 (PDF 57.4 KB) [file 18_2025_5735_MOESM3_ESM.pdf]

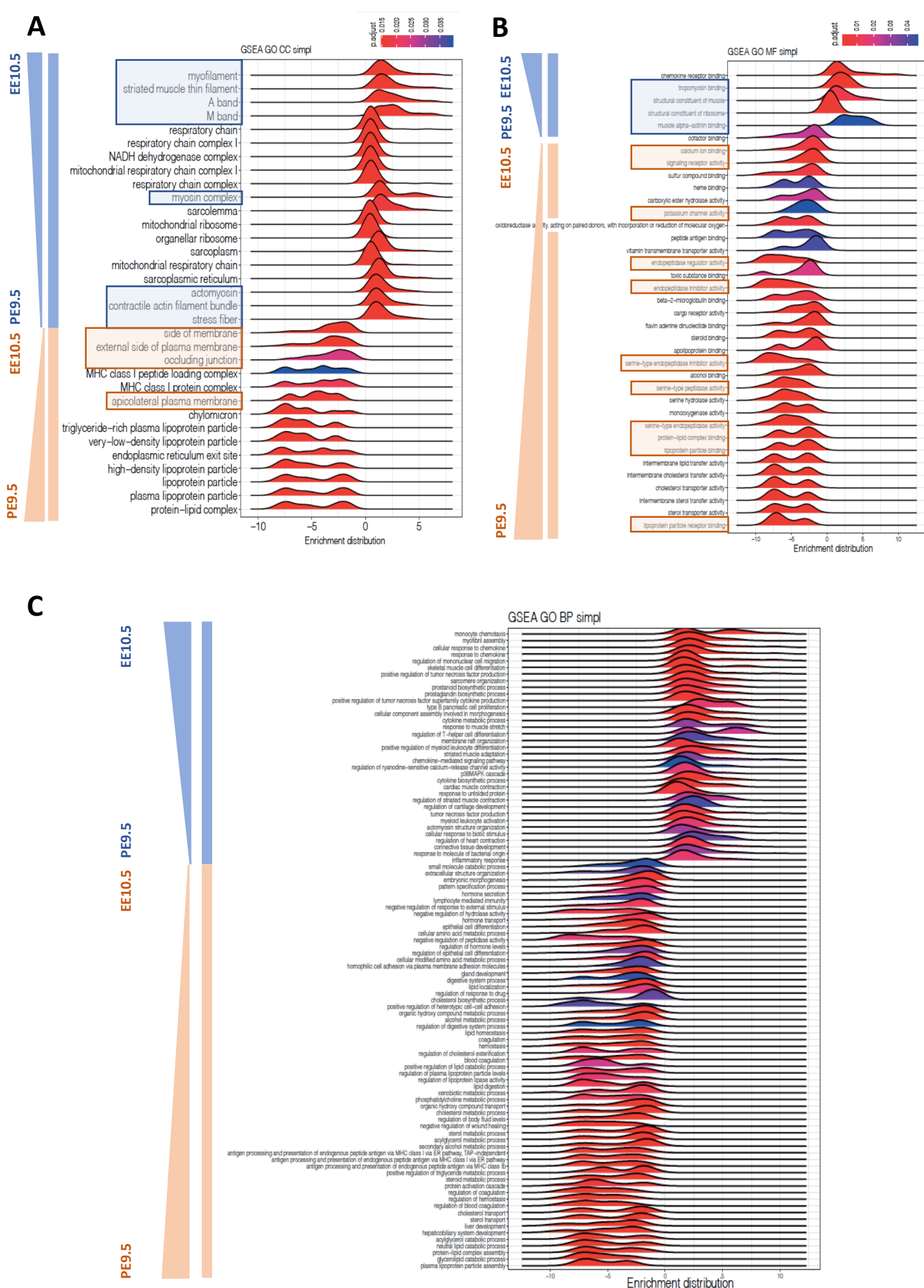

Supplementary Figure 4

Supplement: Supplementary file 4 — Supplementary file4 (PDF 2.13 MB) [file 18_2025_5735_MOESM4_ESM.pdf]

EE10.5

PE9.5

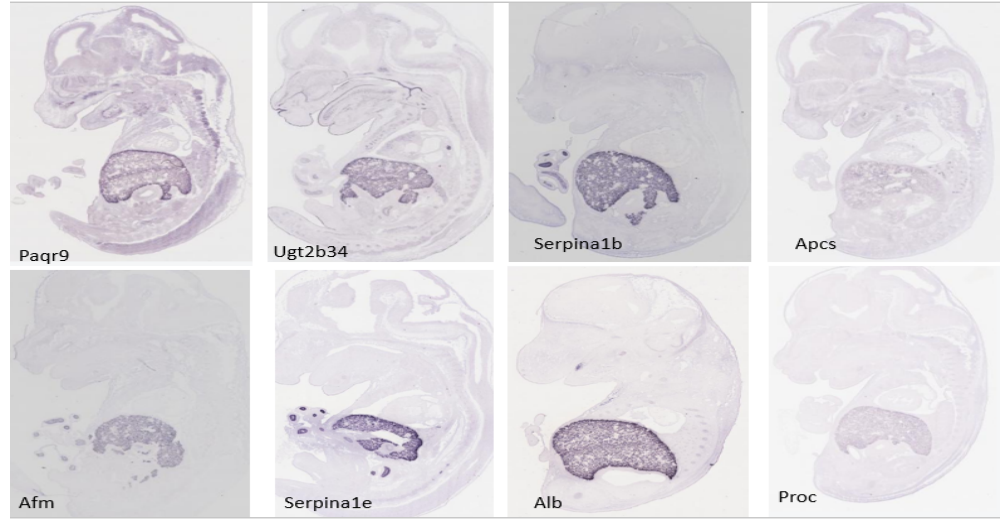

liver

EE10.5

PE9.5

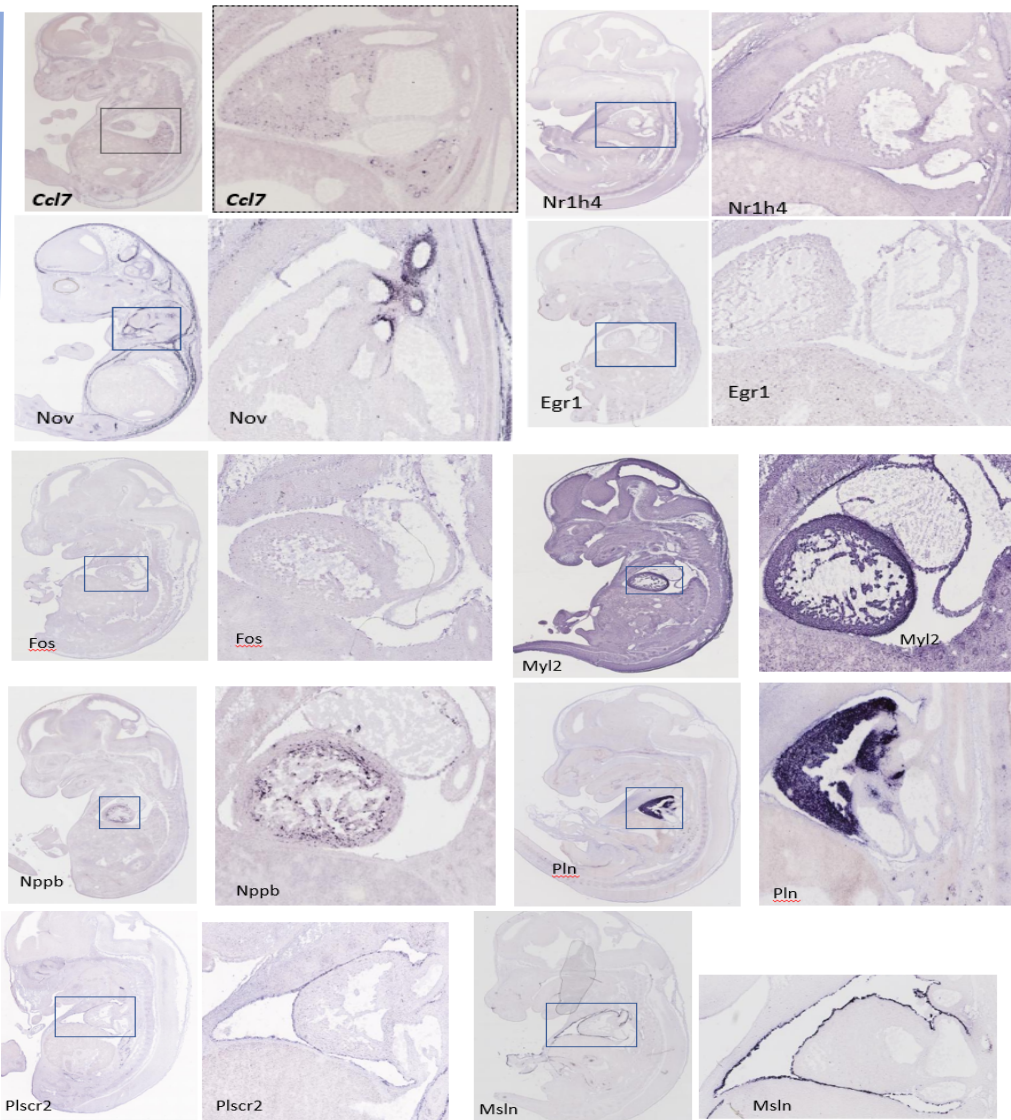

endo

myo

epi

Supplementary Figure 5

Supplement: Supplementary file 5 — Supplementary file5 (PDF 1.86 MB) [file 18_2025_5735_MOESM5_ESM.pdf]

# Biological Theme Comparison

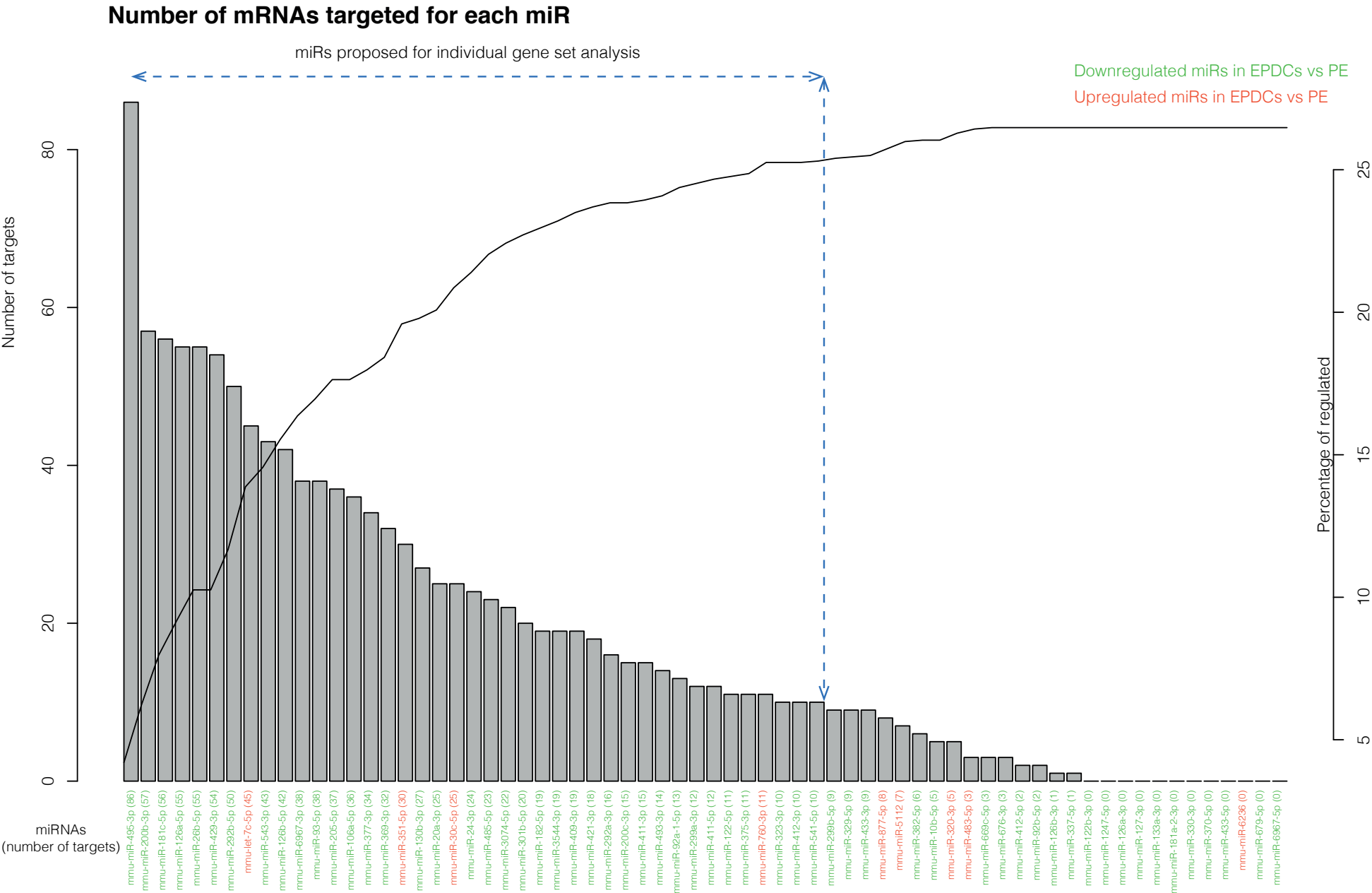

Supplementary Figure 6

Supplement: Supplementary file 6 — Supplementary file6 (PDF 32.7 KB) [file 18_2025_5735_MOESM6_ESM.pdf]

# Cluster comparative MF

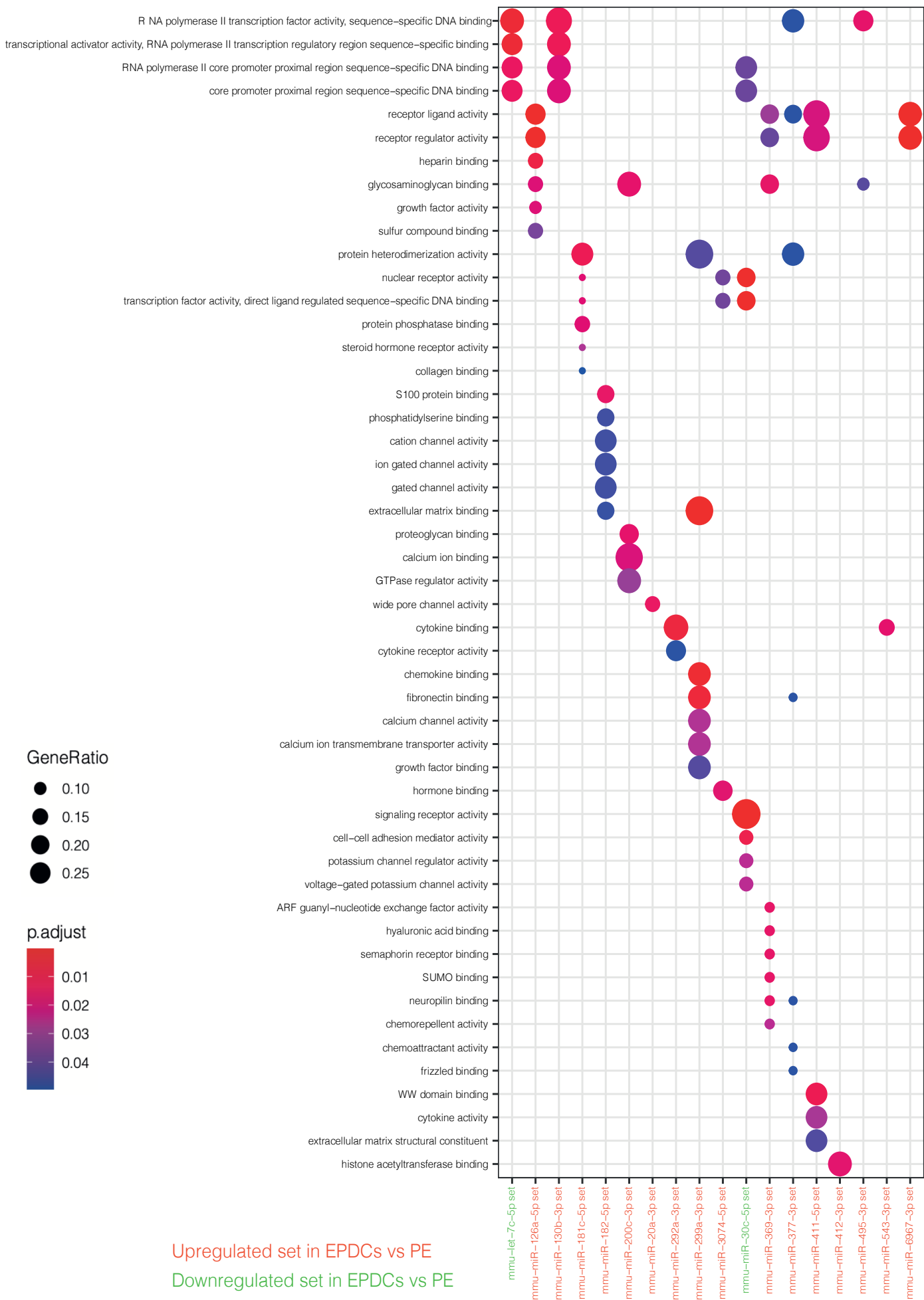

Supplementary Figure 7

Supplement: Supplementary file 7 — Supplementary file7 (PDF 96.4 KB) [file 18_2025_5735_MOESM7_ESM.pdf]

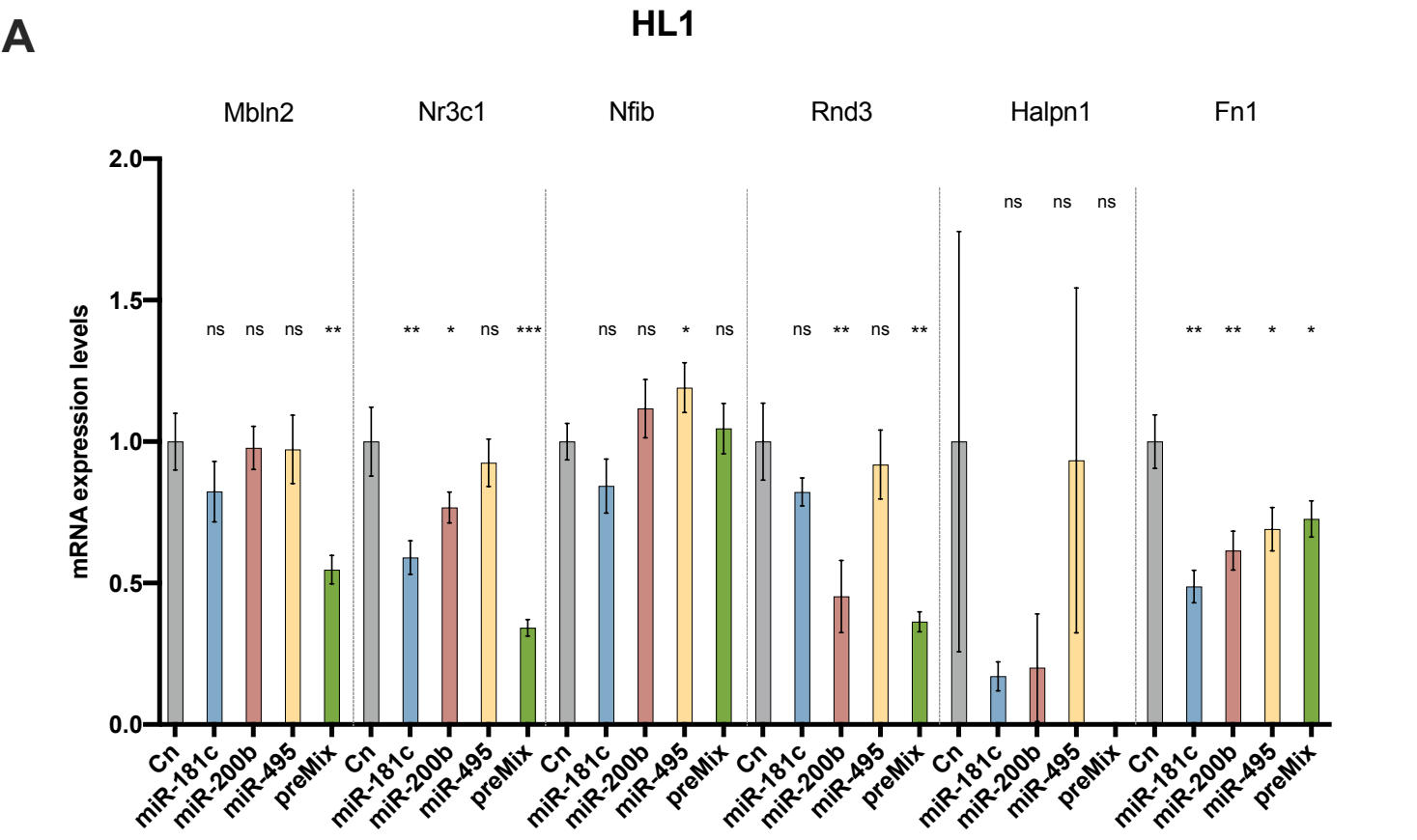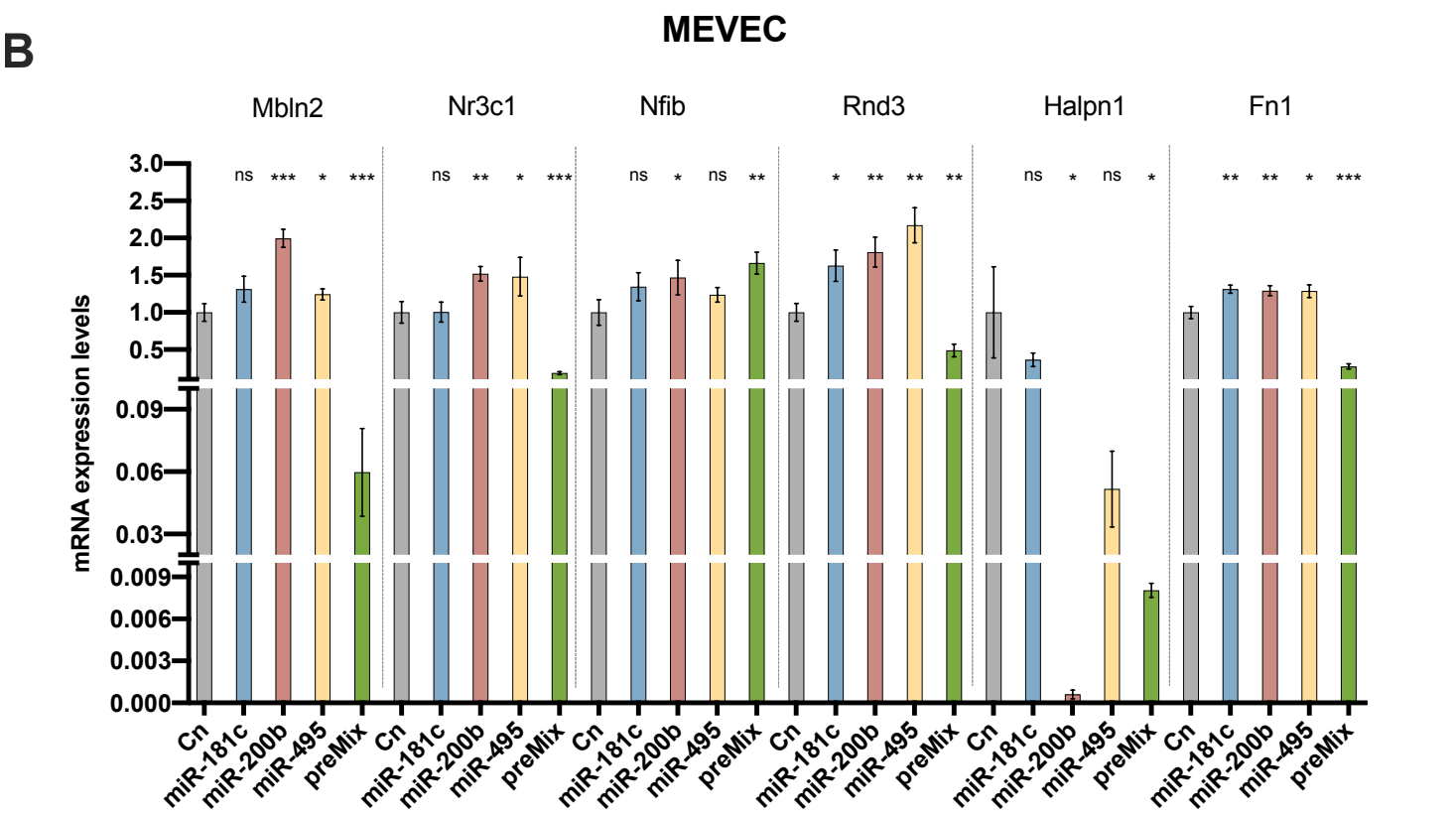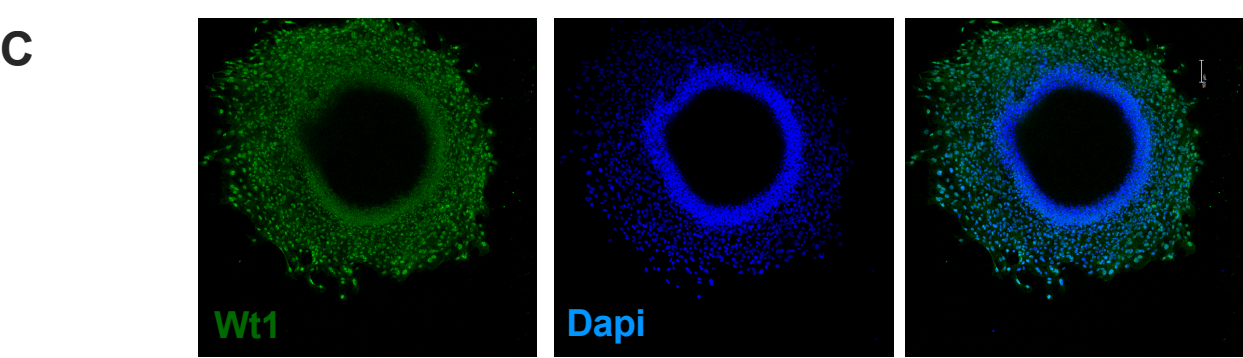

Supplementary Figure 9

Supplement: Supplementary file 9 — Supplementary file9 (PDF 2.25 MB) [file 18_2025_5735_MOESM9_ESM.pdf]

**A**

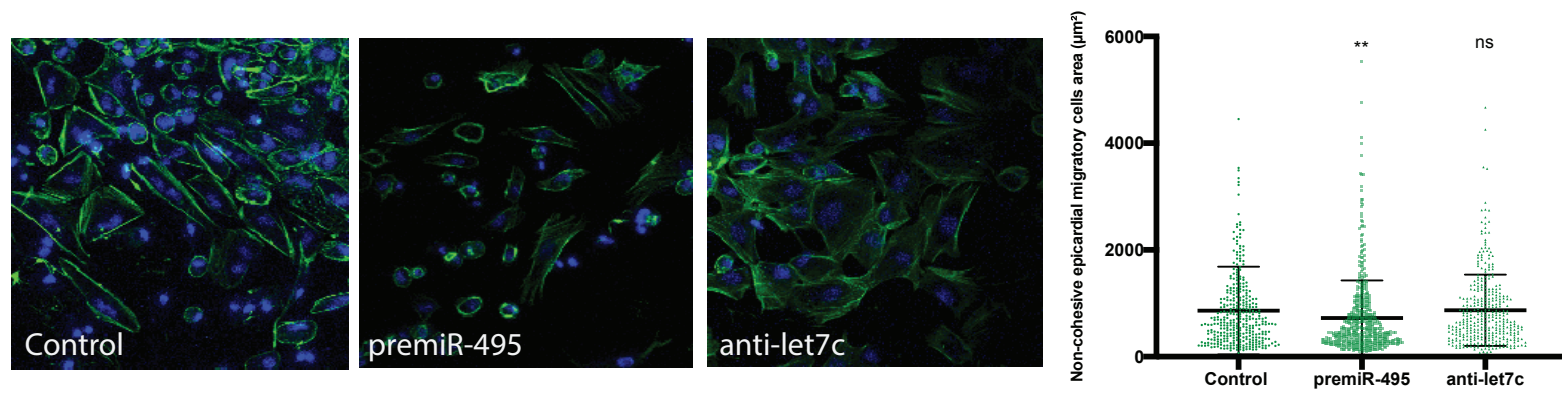

**B**

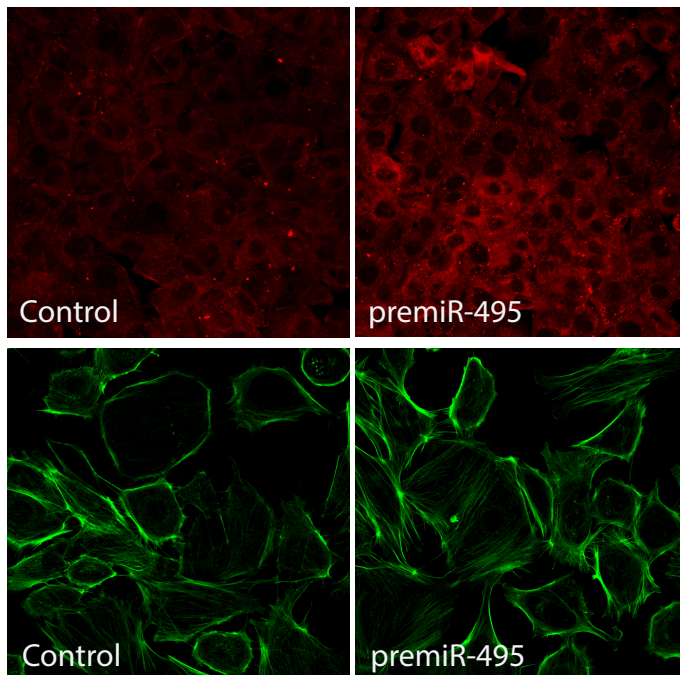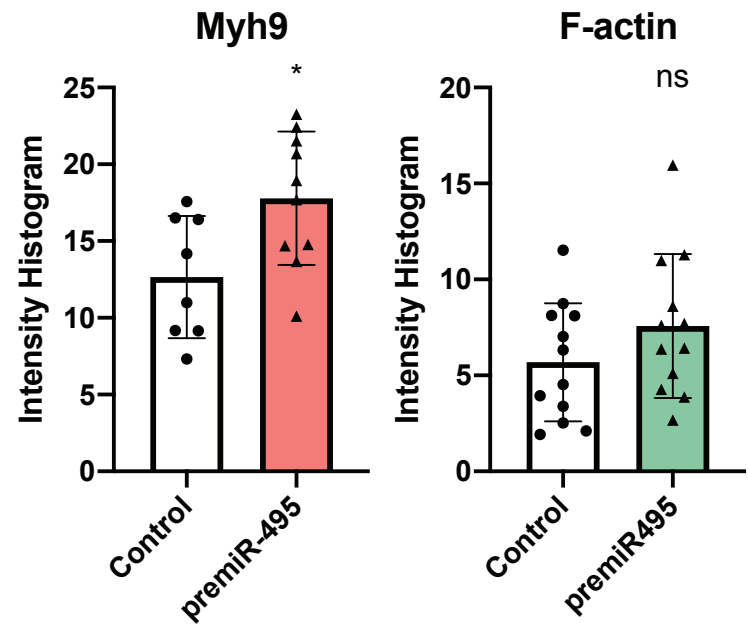

**C**

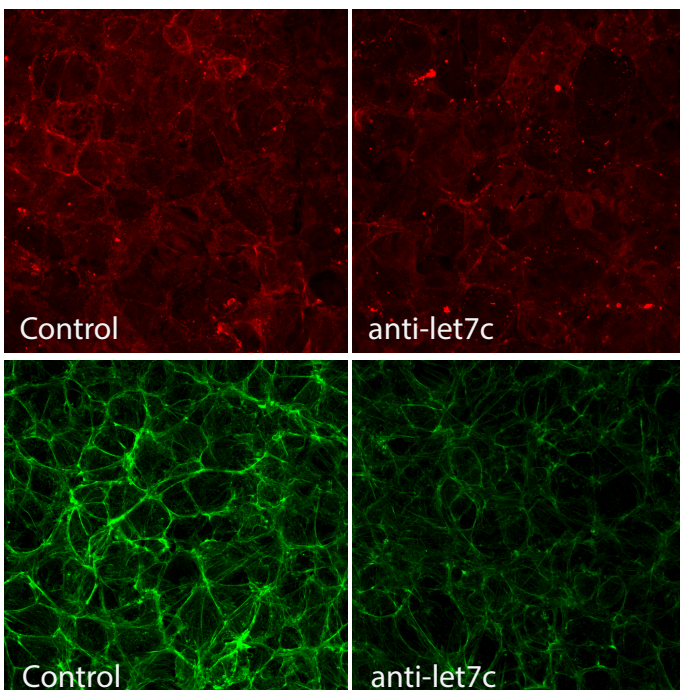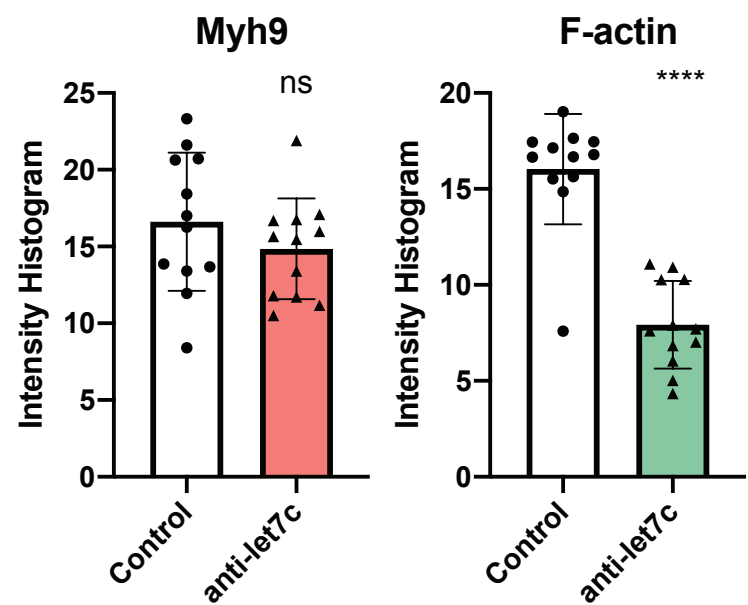

Supplement: Supplementary file 10 — Supplementary file10 (PDF 7.08 MB) [file 18_2025_5735_MOESM10_ESM.pdf]

A

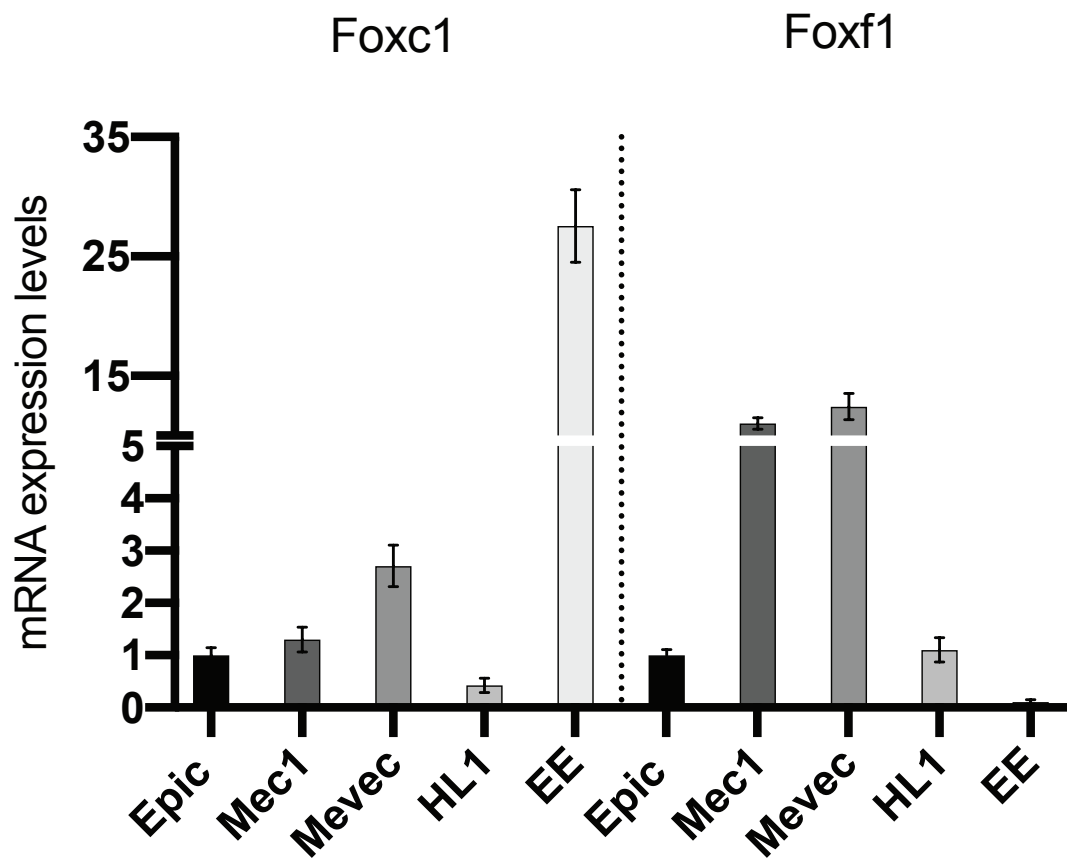

B

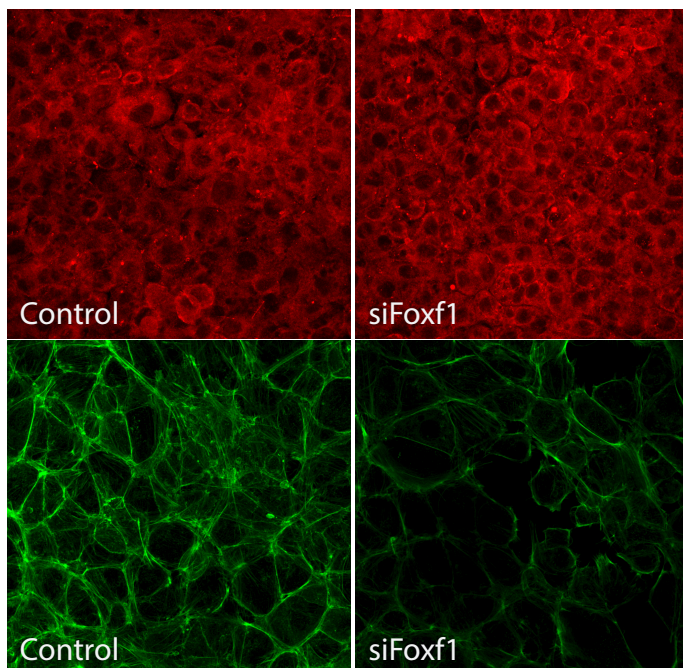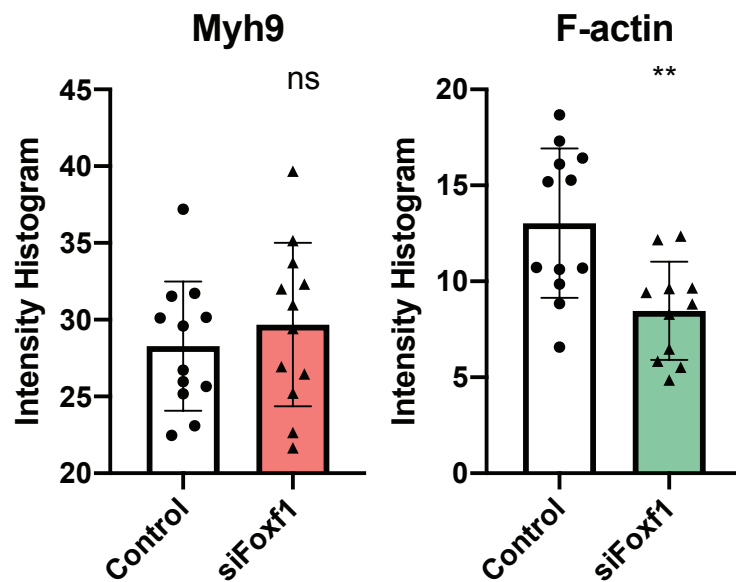

Supplement: Supplementary file 13 — Supplementary file13 (PDF 4.01 MB) [file 18_2025_5735_MOESM13_ESM.pdf]
