# Supplementary material for: Foxf1-mediated co-regulation of miR-495 and let-7c modulates epicardial cell migration and myocardial specification
Source: Cell Mol Life Sci. 2025 Jun 25;82(1):254. doi: 10.1007/s00018-025-05735-4 (PMC12187632; doi:10.1007/s00018-025-05735-4)
Supplement: Supplementary file 8 — Supplementary file8 (PDF 523 KB) [file 18_2025_5735_MOESM8_ESM.pdf]

A

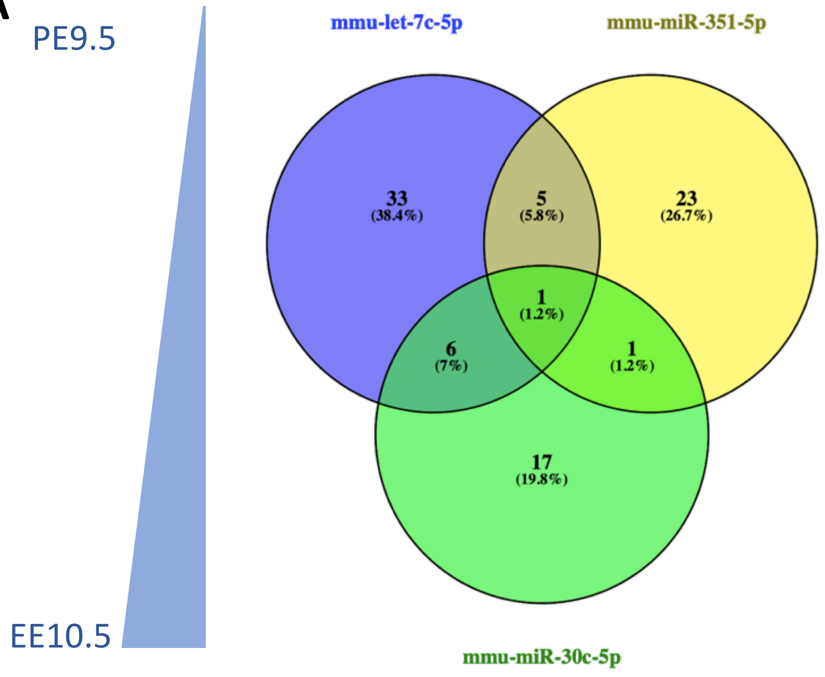

Common mRNA targets PE9.5<EE10.5
**mmu-let7c-5p / mmu-miR-351-5p / mmu-miR-30c-5p (1)**
Prtg
**mmu-let7c-5p / mmu-miR-351-5p (5)**
Sema4c, Lin28a, Scn4b, Trim71, Hic2
**mmu-miR-351-5p / mmu-miR-30c-5p (1)**
Ccnjl
**mmu-let7c-5p / mmu-miR-30c-5p (6)**
Nr6a1, Fign, Mttp, Peg10, Rfx6, Fbxo32

B

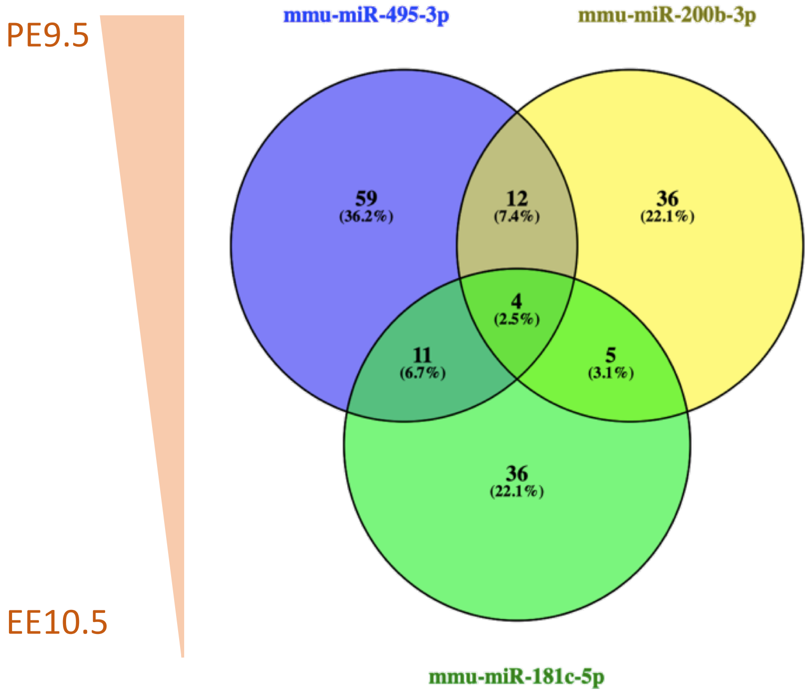

Common mRNA targets PE9.5>EE10.5
**mmu-miR-495-3p / mmu-miR-200b-3p / mmu-miR-181c-5p (4)**
Nfib, Mbln2, Kat2b, Nr3c1
**mmu-miR-495-3p / mmu-miR-200b-3p (12)**
Fn1, Rnd3, Elf2, Rapgef2, Plxna4, Gpm6a, Psd3, Amotl2, Arl4a, Halpn1, Dusp1, Vegfa
**mmu-miR-200b-3p / mmu-miR-181c-5p (5)**
Tsc22d2, Dlg2, 4921524J17Rik, Cpd, Cbx4
**mmu-miR-495-3p / mmu-miR-181c-5p (11)**
Col16a1, Acer3, Sox6, Dmxl,2, Dusp6, Sept8, Gpr22, Akap6, Adamts5, Aqp4, Mcc

Supplementary Figure 8
