# Supplementary material for: Foxf1-mediated co-regulation of miR-495 and let-7c modulates epicardial cell migration and myocardial specification
Source: Cell Mol Life Sci. 2025 Jun 25;82(1):254. doi: 10.1007/s00018-025-05735-4 (PMC12187632; doi:10.1007/s00018-025-05735-4)
Supplement: Supplementary file 11 — Supplementary file11 (PDF 430 KB) [file 18_2025_5735_MOESM11_ESM.pdf]

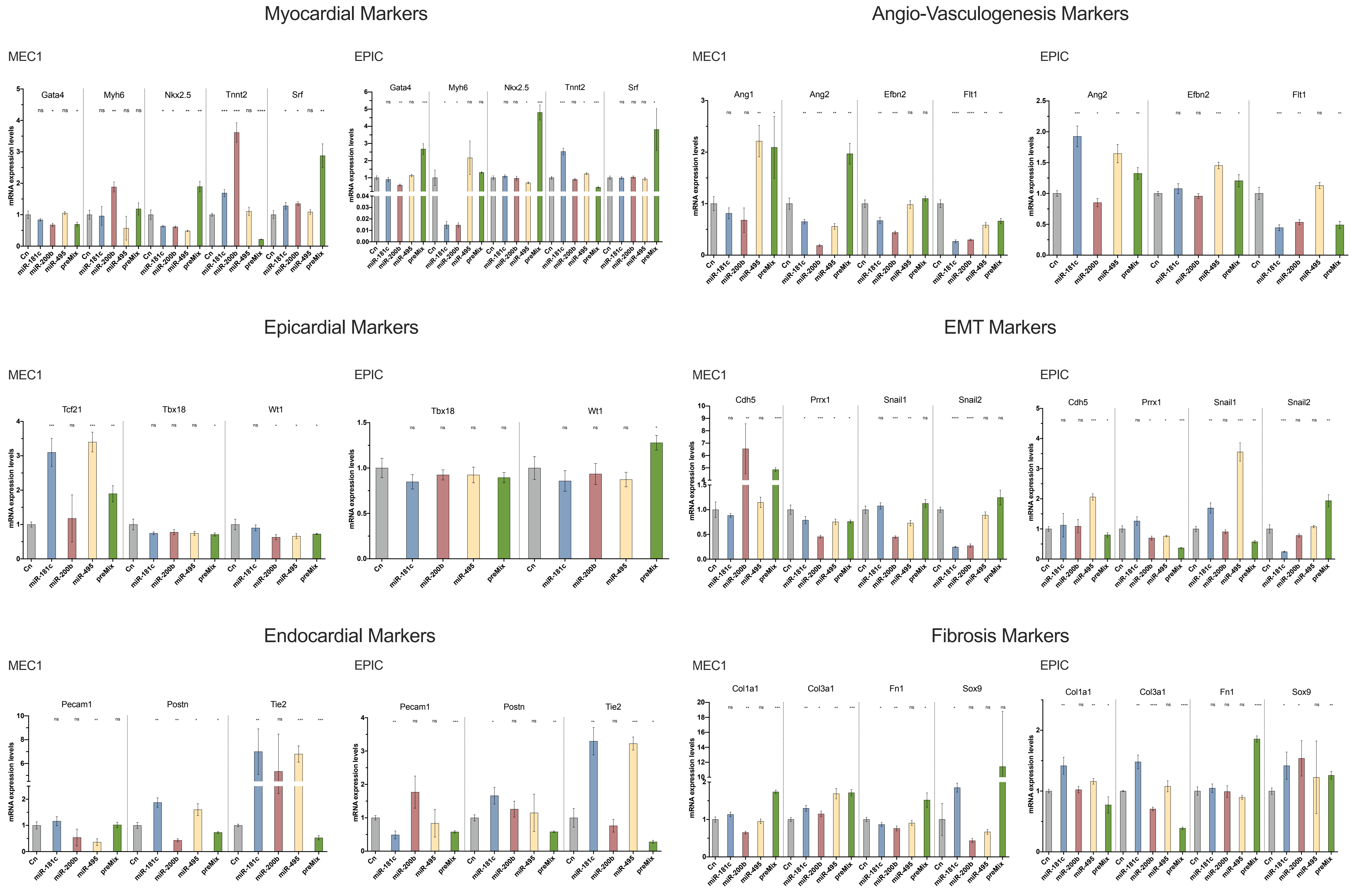

Supplementary figure 11
